# Tranexamic acid in hip and spine surgery for children with cerebral palsy — a PRISMA-compliant scoping review

**DOI:** 10.1186/s13643-024-02734-7

**Published:** 2024-12-27

**Authors:** Daniel Gould, Haoze Cui, Norine Ma, George Chalkiadis, Andrew Davidson, Kerr Graham, Erich Rutz

**Affiliations:** 1 Department of Paediatrics, The Royal Children’s Hospital, University of Melbourne, 50 Flemington Road, Parkville, VIC 3052 Australia; 2 Monash Medical Centre, 246 Clayton Rd, Clayton VIC 3168, Melbourne, Australia; 3 Alfred Health, 55 Commercial Rd, Melbourne VIC 3004, Australia; 4 Department of Anaesthesia and Pain Management, Royal Children’s Hospital, 50 Flemington Road, Parkville VIC 3052, Melbourne, Australia; 5 Department of Orthopaedics, The Royal Children’s Hospital, Melbourne, 3052 Australia; 6 Murdoch Children’s Research Institute, Melbourne, 3052 Australia; 7 Department of Paediatrics, Bob Dickens Chair, Paediatric Orthopaedic Surgery, The University of Melbourne, Melbourne, 3010 Australia; 8 Medical Faculty, University of Basel, Basel, 4001 Switzerland

**Keywords:** Cerebral palsy, Orthopaedics, Tranexamic acid, Blood loss, Transfusion, Scoping review

## Abstract

Many children with cerebral palsy (CP) are frail and require major hip and/or spine surgeries associated with substantial blood loss. Tranexamic acid (TXA) is commonly used to reduce blood loss, but there is uncertainty around the optimal dose and timing of administration. There have been reviews in sub-populations and specific dosing regimens, but a broad overview of the available literature is lacking.

The aim of this review was to map available evidence on TXA in hip and spine surgery for children with CP.

Given the heterogeneous literature, a prospectively registered scoping review was conducted. Eligibility criteria were broad. Three screeners were involved, with the senior author consulted when disagreements were not resolved through discussion.

Titles and abstracts of 14,609 records were screened, with 52 records included. Two additional records were obtained from grey literature and citation searching. Cohort studies (50.0%) were the most common. Most records (76.9%) were on spine surgery. TXA dose varied widely. Loading doses range from 5 to 100 mg/kg and intraoperative infusions from 1 to 10 mg/kg/h. Dose was not reported in 35.2% of records. Primary outcome measures included blood loss and transfusion requirements. TXA was generally reported to be safe. None of the included records reported postoperative TXA administration.

While TXA is generally considered safe, there was mixed evidence on efficacy. Much of the evidence was drawn from studies in which TXA was used in patients at higher risk of bleeding or with reduced physiological reserve. There was no evidence for TXA being used postoperatively, when a large proportion of transfusions occur.

**Supplementary Information:**

The online version contains supplementary material available at 10.1186/s13643-024-02734-7.

## Introduction

Cerebral palsy (CP) occurs at a rate of 2.11 per 1000 live births worldwide [1], with a higher prevalence in low- and middle-income countries where birth rates are highest [2]. It has been identified as one of the leading causes of childhood disability worldwide [3]. Approximately 35% of children with CP experience clinically significant hip displacement [4, 5], and 15–80% develop scoliosis [6, 7]. Hip and spine deformities can cause pain and functional limitations rendering surgery as the only viable management option [4, 6, 8], and these deformities frequently occur simultaneously [9]. The benefits of bony reconstructive surgery are well-documented for children with cerebral palsy across all levels of the Gross Motor Function Classification System (GMFCS). [10] Surgical goals may include improvements in gait at GMFCS Levels I-III and comfortable sitting and relief of pain for those with more severe limitations. Complications are common and may affect between 25 and 80% of children, which may prolong the length of stay and increase the likelihood of hospital readmission [11–13]. Deciding when to operate and to whom surgery should be offered as a management option is a complex shared decision between patients, their families and carers, and the multidisciplinary treatment team. A major challenge in the decision-making process stems from the fact that children who might be candidates for this type of surgery have a very high prevalence of medical comorbidities which increases the risk of postoperative complications [4]. Specifically, these procedures are associated with a substantial degree of blood loss [6, 14]. Perioperative bleeding, even below the level at which transfusion of blood products is indicated, is a risk factor for postoperative complications [15]. Bleeding can often be so severe that blood transfusion is required, and in paediatric surgical populations, the presence of CP itself is a risk factor for requiring transfusion [16–18]. Transfusion carries the risk of further complications, including blood type incompatibility, lung injury and circulatory overload, and there is evidence that these complications may be underreported in paediatric surgical populations [19]. Furthermore, reducing transfusion rate is an important aspect of global surgery considering the relative scarcity of this resource in many parts of the world [20].

Tranexamic acid (TXA) is an antifibrinolytic agent that prevents the breakdown of blood clots which reduce perioperative blood loss [21]. Designated as one of the World Health Organization’s Essential Medications [22], TXA is particularly useful in major surgery for children with CP, given that around one third of children with CP have epilepsy [23] and commonly used antiepileptic medications lead to increased perioperative bleeding [24]. However, the use of TXA is not without its risk, and careful consideration must be taken to balance the risk of seizure associated with its use [25]. There is mixed evidence on the efficacy of TXA in this patient population despite it being commonly used [26], and little consensus on optimal dosing regimens with recent reviews limited to specific study designs [27–30], specific TXA dosing regimens [31], specific modes of delivery of TXA [32], or a specific type of surgery in order to conduct meta-analysis [33]. The literature lacks a comprehensive systematic exploration of the use of TXA in hip and spine surgery for children with CP to capture the state of knowledge on this topic, facilitate the identification of knowledge gaps and lay the foundation to address these gaps by informing the design of rigorous clinical trials [34]. In this context, a scoping review is crucial, as its methodology is particularly well-suited to summarise the literature in this way [35].

The objective of this review was to systematically and comprehensively map the literature pertaining to the use of TXA in hip and spine surgery for children with CP, to better understand the following characteristics of the literature: types of studies conducted, outcomes measured, methods of outcome measurement, dosing regimens of TXA, and the positive and negative impacts of TXA on outcomes.

## Methods

### Protocol and registration

The protocol for this scoping review was developed in line with the PRISMA (Preferred Reporting Items for Systematic reviews and Meta-Analyses) extension for scoping reviews, PRISMA-ScR [36], and prospectively registered with Open Science Framework (link: https://osf.io/uhj4a/?view_only=17876ae9bbf04ed6bf15d95d87559e17).

Review question: What is the impact of TXA on blood loss during hip and spine surgeries for paediatric patients with cerebral palsy?

### PICO (eligibility criteria)

Population: paediatric patients with CP undergoing hip or spine surgery.

- ‘Neuromuscular’ was also included, even if CP was not specified.
- ‘Hip dysplasia’, not further specified, was also included when neuromuscular hip dysplasia was not excluded from the study population.
- All functional levels were included. In CP literature this is typically measured using the Gross Motor Function Classification System (GMFCS)
- Exclusions: non-human animal studies, trauma and hip fracture, and arthroplasty Intervention: perioperative administration of TXA.
- ‘Antifibrinolytic’ was also included, provided the study did not specifically mention that TXA was not used

Comparison: placebo, no TXA, different antifibrinolytic agent, or different dosing regimen of TXA.

Outcomes: blood loss was the primary outcome of interest. Transfusion rate, length of stay, and complication rates were also collected.

### Information sources

The primary search strategy was constructed in line with guidelines on optimal database searching [37] and in conjunction with an experienced librarian. This comprised a search initially generated and refined in MEDLINE (Ovid). This was then adapted to EMBASE (Ovid), Web of Science Core Collection, and then to Google Scholar (advanced search functionality) from which the first 200 results were retrieved. No limitation was placed on the type nor year of publication. There was also no limitation on study design, given that studies which precluded inference regarding the impact of TXA could still be useful in terms of ascertaining the way in which TXA is reported in the literature and the variety of dosing regimens that have been used in this patient population.

This primary search strategy was supplemented by a grey literature search as well as forward and backward citation searching.

The full search strategy is outlined in the subsection, ‘Search strategy’.

The information sources searched as part of the grey literature search strategy were the following: WHO ICTRP (World Health Organization Clinical Trials Registry Platform), ClinicalTrials.gov, New York Academy of Medicine Grey Literature Report, Open Science Framework, ProQuest, Dimensions, Trove, WHO IRIS (World Health Organization Institutional Repository for Information Sharing), and a general Google search.

### Search strategy

The search was broad to capture orthopaedic and spine surgery, with studies of paediatric CP populations being selected during the screening process. The search strategy, developed in collaboration with the librarian, was constructed in Medline (Ovid). It was tested and modified in multiple iterations to arrive at the final strategy which had sufficient sensitivity to ensure relevant records were identified for data charting. The strategy was then adapted to Embase, Web of Science Core Collection, and Google Scholar using the advanced search functionality. There were two key concepts, ‘tranexamic acid’ and ‘orthopaedics’. The Supplementary File section, ‘Search strategies’, contains the full search strategy for each database. The grey literature search strategy is detailed in the Supplementary File, Table S1.

Forward and backward citation-chasing was carried out on the final collection of included articles after grey literature search was completed. CitationChaser [38] was used as part of this process, and the list of articles returned from this process was screened using the same criteria as for the screening process of records identified from the primary literature search. Given that CitationChaser relies on DOI (digital object identifier), author KC manually conducted forward and backward citation searching for records that did not have a DOI.

### Selection of sources of evidence

Records identified from the primary literature search were imported into Covidence software and de-duplicated. Three reviewers (DG, KC, and NM) screened titles and abstracts, with each record requiring two screeners agreeing on the decision to include or exclude. The screening process was pilot tested through four iterations to finalise the guidance document used by the reviewers to ensure the eligibility criteria were clearly understood and applied consistently throughout the screening process. Disagreements were resolved through discussion. The senior author (ER) was available to be consulted in cases of disagreement which could not be resolved through discussion.

Full-text screening was carried out in the same way, with a separate guidance document developed through two iterations of pilot testing.

After the completion of full-text screening and data charting, the grey literature search was run by authors DG and KC. Screening of records was carried out by author KC and verified by author DG. Author KC then carried out forward and backward citation chasing of all included records. The same eligibility criteria as the primary search were applied and data charting was carried out in the same way.

Automated screening tools were not utilised in this review.

### Data charting process

The data charting form was developed in Microsoft Excel with input from authors DG, KC, NM, and ER to ensure data relevant to the research question were extracted. It was pilot tested with KC and NM, each of whom extracted data from five separate studies. Author DG extracted data from these 10 studies and then refined the data charting form in collaboration with authors KC and NM accordingly to ensure additional relevant information was collected and all variables were clearly defined. DG then extracted data from the remaining included studies.

### Data items

The Supplementary File, Tables S2 to S9, contain the full list of extracted variables. Tables S2 and S3 contain information pertaining to study characteristics: type of record, study design, study setting and the period over which it took place, patient population and surgical procedure, and primary study aim. The study design was determined based on the use of TXA. For example, a study was considered to be of case series design for the purpose of this review if all patients received TXA with no difference in the dosing regimen. Tables S4 and S5 contain information pertaining to characteristics of patients and TXA dosing reported in included studies: age, sex, functional level, TXA dosing regimen, primary outcome measure, and sample size calculation. Tables S6 and S7 contain information pertaining to study findings for studies with a primary aim related to TXA: blood loss measurement technique, funding source, findings pertaining to blood loss and transfusion, and findings related to complications. Tables S8 and S9 contain the same information for studies with a primary aim not related to TXA.

### Critical appraisal of individual sources of evidence

In line with scoping review methodological guidelines, this was not carried out [34].

### Synthesis of results

In line with the objective of this review, characteristics of the included records were summarised descriptively in tabular format such that the current state of the evidence on this topic could be better understood. Next, information pertaining to the patients, intervention, and outcomes was summarised to understand the patient populations in which TXA is used as well as the variability in the dosing regimens and reporting of TXA in these populations. Figures were generated to summarise the findings on blood loss and transfusion from to studies with a primary aim directly related to TXA. The findings of studies with a primary aim not directly related to TXA were summarised in tabular format. This decision was made because the search strategy and eligibility criteria were broad, therefore it was deemed likely that a substantial proportion of records with a primary aim not related to TXA would contain information on TXA without sufficient detail to determine its impact. Data on the safety of TXA and its use in clinical practice for this patient population were then summarised descriptively.

## Results

### Selection of sources of evidence

**Fig. 1:**
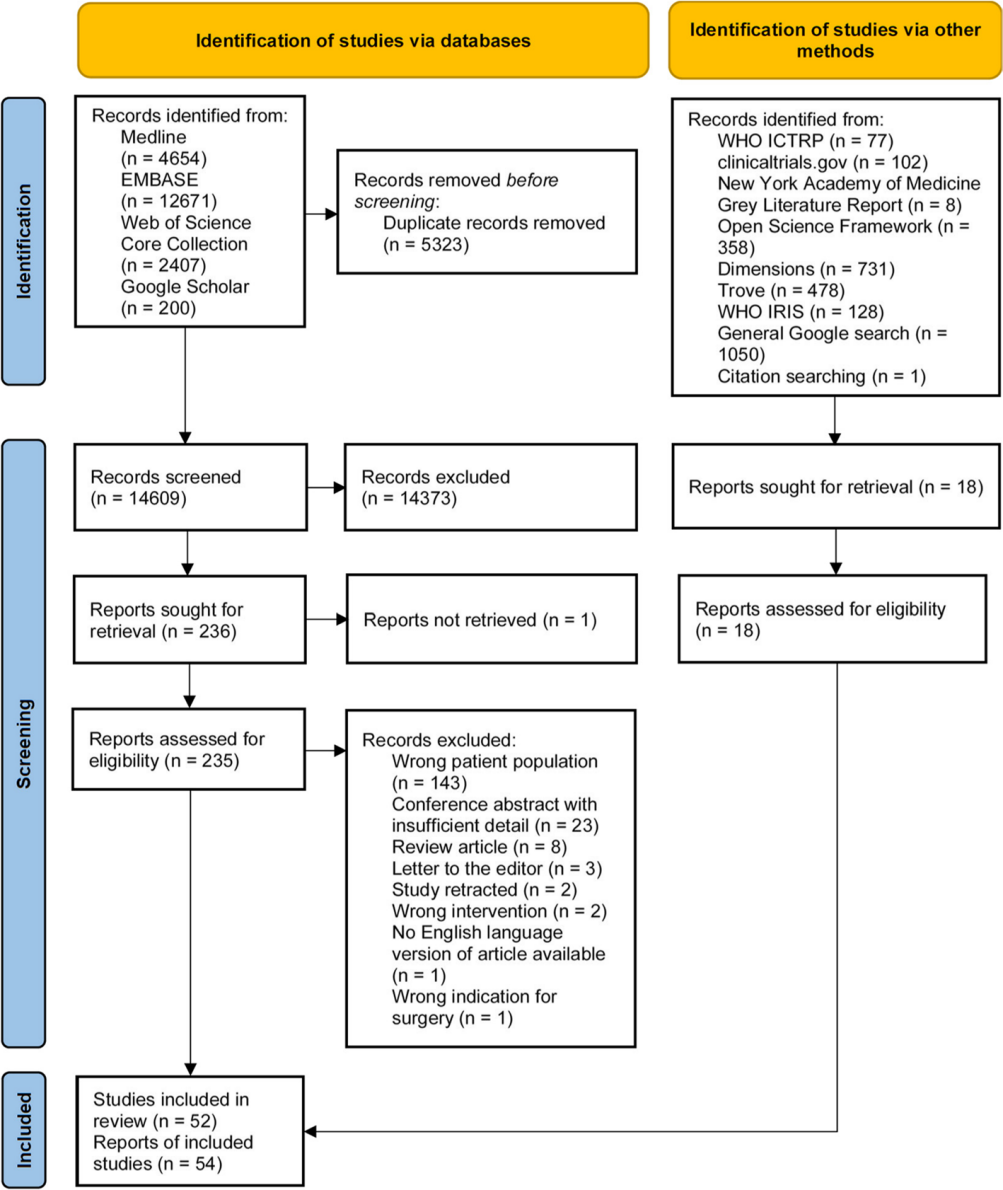
PRISMA flow diagram

The primary literature search was run on 13 October 2023. Grey literature and citation searching were carried out between 16 and 18 May 2024. The outcomes of the search and screening processes are outlined in Fig. 1, which depicts a PRISMA flow diagram [39]. Table S10 depicts the findings of grey literature searching which resulted in the inclusion of two additional records [40, 41]. Citation chasing did not result in the inclusion of any additional records (Fig. 1).

One record reported on the same study as Dupuis et al. [42]. Another record was a conference abstract for the study which was later published by Lins et al. [43]. Neither of these records provided any additional information, therefore they were excluded from data charting (Table 1)

**Table 1:** Characteristics of included records

**Characteristic**	**Findings **
Type of study (*n* (% of total))	Cohort study = 26 (50.0%); case series = 13 (25.0%); **RCT = 4 (7.7%); ***RCT protocol = 3 (5.8%); case report = 2 (3.8%); unclear = 2 (3.9%); case control = 1 (1.9%); survey = 1 (1.9%)
Study setting (*n* (% of total))	Single-centre = 44 (84.6%); multi-centre = 8 (15.4%)
Study population (*n* (% of total))	Spine = 40 (76.9%); hip = 11 (21.2%); mixed (hip and spine) = 1 (1.9%)
Sample size (median (range))	41.0 (1 to 830) patients with CP or neuromuscular condition
*Proportion of total sample size comprising patients with CP	Among studies with proportion (%) of CP reported (mean (range)) = 59.3% (1.7 to 100%) of 18 records Proportion with neuromuscular disorder in papers which reported this instead of CP (mean (range)) = 31.3% (5.8 to 62.5%) of 19 records Neither CP nor neuromuscular disorder specified (n (% of total)) = 14 (26.9%) of 52 records

*CP *cerebral palsy; *excluding a survey study but including the planned sample size of RCT protocols; **no indication of prospective registration of any of these trials; ***all prospectively registered

Cohort studies comprised the largest proportion of studies by design. Most studies were single-centre and the majority were on patients undergoing spine surgery. The median number of patients with CP was 41, comprising a widely varied proportion of the total number of participants in the included studies.

Table S2 contains these details for each individual study. Additional summary information is contained in Table S3.

### Characteristics of patients and interventions

Table 2 contains information on the characteristics of the study populations and dosing regimens of TXA they received (Table 2).

**Table 2:** Characteristics of patients and interventions in included records

Characteristic	Findings—spine (*n* = 41 studies)	Findings—hip (*n* = 12 studies)
Age (years)	Range of reported mean values = 9.9 to 41.4 Not reported = 6 (14.6%)	Range of mean values = 6.5 to 11.1 Not reported = 1 (8.3%)
Sex	Range of proportions (% female) = 38.6 to 89.2 Not reported = 12 (29.3%)	Range of proportions (% female) = 35.0 to 69.1 Not reported = 0
Functional level	GMFCS level = 2 studies (4.9%) Wheelchair dependence = 1 study (2.4%) Spastic quadriplegia (not further specified) = 1 study (2.4%) SRS-24 = 1 study (2.4%) Not reported = 36 (87.8%)	GMFCS level (range of proportions of patients in each category): - GMFCS I = 1.3% to 3.2% - GMFCS II = 4.0% to 31.3% - GMFCS III = 11.1% to 38.5% - GMFCS IV = 21.4% to 56.3% - GMFCS V = 0 to 64.5% Not reported = 4 (33.3%)
TXA dosing	Number of studies using bolus only = 1 (2.4%) Number of studies using infusion only = 0 Number of studies using bolus and infusion = 23 (56.1%) Range of bolus dose (mg/kg) = 5 to 100 Range of intraoperative infusion dose (mg/kg/h) = 1 to 10 Number of studies with unclear or insufficient reporting = 17 (41.5%)	Number of studies using bolus only = *3 (25.0%) Number of studies using infusion only = 0 Number of studies using bolus and infusion = *7 (58.3%) Range of bolus doses (mg/kg) = 10 to 50 Range of intraoperative infusion dose (mg/kg/h) = 1 to 10 Number of studies with unclear or insufficient reporting = 3 (25.0%)

TXA given intravenously unless otherwise specified; *GMFCS*, Gross Motor Function Classification System; *SRS-24*, Scoliosis Research Society 24-item questionnaire; *one study included a bolus-only arm and a bolus + infusion arm

Age was more varied among the spine studies than in the hip studies. The proportion of female patients varied, being the majority in some studies and minority in others. Functional level was reported in a minority of spine studies, whereas it was reported much more consistently in hip studies where the GMFCS was used in 66.7% of studies. TXA dosing was inconsistently reported and varied more widely in spine studies than in hip studies; however, the most frequent dosing regimen was a preoperative bolus followed by intraoperative infusion. The intraoperative infusion dosing was similar between hip and spine studies.

Table S4 contains these details for each individual study. Additional summary information is contained in Table S5.

### Efficacy

**Fig. 2:**
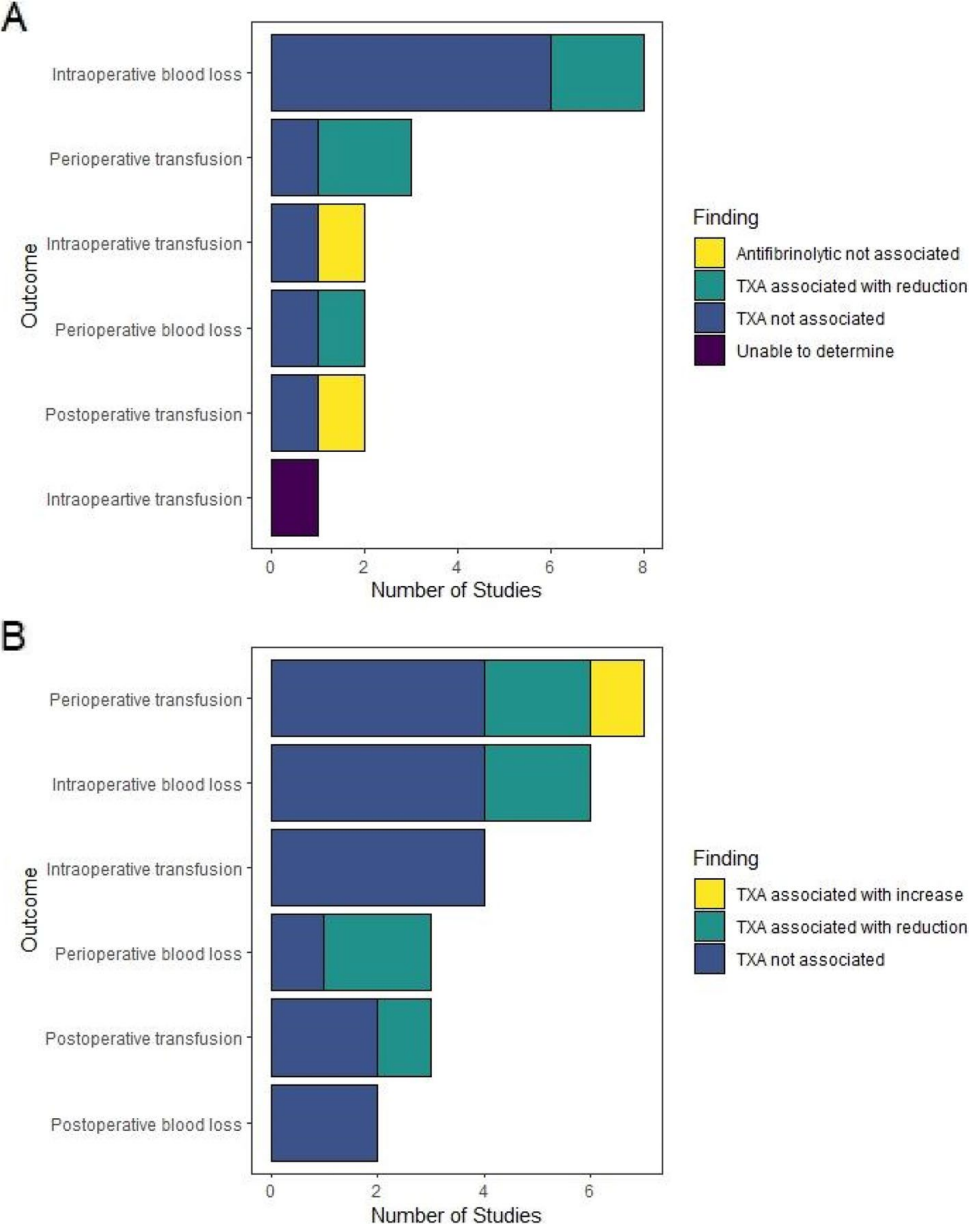
Findings pertaining to the impact of TXA on blood loss

Figure 2 summarises findings pertaining to the impact of TXA on blood loss and transfusion for studies with a primary aim directly related to TXA in spine and hip surgery patients, respectively. Tables S6 and S7 contain detailed information on the data used to produce these figures. These supplementary tables also contain details on the different ways in which blood loss was measured in the included studies (Fig. 2).

This figure demonstrates that intraoperative blood loss was the most commonly reported outcome. Findings were mixed regarding the impact of TXA on reducing transfusion rate and blood loss throughout the perioperative period.

TXA was found to be associated with an increased perioperative transfusion rate in one study. This study [44] was one of six retrospective studies [43, 45–48] included in this review which specified that TXA was used in patients with more severe deformities undergoing more extensive surgery, representing a major confounding factor when interpreting the independent effect of TXA on outcomes. Table S4 contains the details of each of these studies, including the patterns of TXA dosing. One study measured intraoperative transfusion, but this was a case series therefore it was not possible to determine whether TXA influenced this outcome.

Table S9 contains a summary of findings from studies with a primary aim not directly related to TXA. Neither of the hip surgery studies in this Table measured blood loss nor transfusion. The remaining 29 studies were in spine surgery patient populations and findings were similarly mixed. An association between TXA and outcomes was unable to be determined in the majority of these studies due to case series design or insufficient information.

### Safety

Tables S6 and S9 contain information on complication rates from the minority of studies which reported such data, mostly as secondary outcomes. Six spine surgery studies [47, 49–53] and six hip surgery studies [43, 46, 54–57] reported no increase in complication rate, most commonly in terms of venous thromboembolism, associated with the use of TXA.

Beginning in 2018, there have been six studies published in which TXA was used as part of multimodal perioperative blood loss or transfusion reduction protocols [17, 40, 58–61].

## Discussion

### Summary of evidence

The objective of this review was to systematically and comprehensively map the literature pertaining to the use of TXA in hip surgery and spine surgery for children with CP.

This review found that 60% of included studies (31 of 52 records) did not have a primary aim relevant to TXA administration. Figure S1 demonstrates that there has been an increase in the number of studies with a primary aim directly related to TXA in recent years, possibly indicating a growing interest in ascertaining the treatment effect of TXA in this population (Fig. S1).

The majority of the literature on this topic was on spine surgery; however, the number of hip and spine surgery studies with a primary aim directly related to TXA was similar (Table S2).

TXA dosing regimens, primary outcome measures, and measurement techniques for blood loss varied substantially and were reported inconsistently. This poses a challenge in interpreting the literature to determine the impact of TXA in this clinical setting. Considering the fact that TXA is a pharmacological agent administered to reduce blood loss, and that blood loss is known to be difficult to quantify or estimate accurately [62], it is pertinent to draw attention to the fact that many of the included records provided little detail on the method by which blood loss was measured (Tables S7 and S9), or used a technique such as estimation based on soaked gauze and suction output which is known to be inaccurate and unreliable [62]. While there is no gold standard for blood loss measurement technique, there is evidence that haemoglobin-based formulae accounting for transfused blood are more accurate and reliable [63, 64]. There were also cases in which blood loss was only measured using a more accurate measurement technique in cases where blood loss was expected to be higher than average [46].

Confounding factors were identified which influence the interpretation of findings pertaining to the effectiveness of TXA, in that it was used in patients with more severe deformities undergoing more extensive surgery, who also had a greater comorbidity burden [43–48]. This is consistent with prior literature [65].

It is pertinent to the interpretation of findings that Fig. 2 is not viewed in terms of ‘vote counting’ to determine whether TXA was associated with reported outcomes [66]. Rather, it is a visual depiction of the heterogeneity of outcomes and findings. Despite mixed findings on efficacy and limited data on safety of TXA, it appears that TXA is increasingly considered to be safe and effective and is therefore widely used in this clinical population. This is evidenced by recent studies incorporating TXA in multimodal blood loss and transfusion reduction protocols [43, 46, 47, 49–57].

None of the included studies reported utilising TXA in the postoperative period, during which a substantial degree of blood loss [67, 68] and transfusion occur [69].

### Contribution to the literature

This is the first review to employ a broad search strategy to capture the diversity of findings in the literature on the use of TXA in children with CP undergoing hip or spine reconstructive surgery. It demonstrates that there is a lack of high-quality evidence from clinical trials isolating the treatment effect of TXA in this patient population. However, it appears this may reflect the fact that TXA is generally considered to be safe and effective and is therefore routinely being used in clinical practice in accordance with expert opinion [70].

### Implications for future research

Although there are few high-quality randomised controlled trials demonstrating the effectiveness of TXA in reducing blood loss in patients with cerebral palsy undergoing spine or hip surgery, it appears as though it is being widely used in clinical practice. There are trials currently underway in both spine [71] and hip [72] surgery patients investigating the efficacy and safety of TXA administered in the operative period. However, a major evidence gap is on the use of TXA in the postoperative period. There is growing evidence that hidden blood loss occurring in the postoperative period is substantial [68] and can potentially be attenuated with the administration of TXA [73–77]. However, these trials on postoperative TXA have been carried out exclusively in adult surgical populations, presenting an opportunity for novel trials in paediatric populations.

### Strengths and limitations

This review was prospectively registered and conducted in line with up-to-date methodological guidelines [36]. A broad literature search was conducted in accordance with literature on optimal database searching [37]. In addition to this, a comprehensive dedicated grey literature search was carried out as well as forward and backward citation searching on all included records. This broad approach generated a detailed map of the evidence for this commonly used drug in the vulnerable population of children with CP undergoing major bony reconstructive surgery. It also identified a major gap in the literature regarding postoperative administration of TXA. A large proportion of perioperative blood loss occurs postoperatively [67, 68] and the benefit of TXA needs to be weighed against seizure risk [78] in this population that is already prone to seizures [79] and commonly receiving antiepileptic medications which further increase bleeding [24].

The heterogeneity of the literature precluded quantitative synthesis of findings, representing a major limitation in attempting to determine the optimal dosing regimen of TXA to maximise efficacy while minimising complications. Non-English language articles were also excluded; however, this is expected to have a minor impact on findings considering one record was excluded for this reason. Another limitation of this review was that it focused on TXA rather than all antifibrinolytic agents, therefore limiting the capacity to draw inferences regarding the relative impact of TXA compared with other antifibrinolytic agents in this patient population.

## Conclusions

In this scoping review, a broad search retrieved over 17,000 records, of which 52 were included. There were mixed findings on TXA dosing regimens and their impact in hip and spine surgery for paediatric patients with cerebral palsy in reducing perioperative blood loss and transfusion rate. There was evidence that, in the past, TXA was used in patients undergoing more extensive surgeries or for patients with a high comorbidity burden. Furthermore, important details pertaining to dosing regimens and outcome measures were reported inconsistently and incompletely. Despite this lack of clarity, it appears that TXA is generally accepted to be safe and effective and is widely used intraoperatively. This review lays the foundation for further exploration into the potential for TXA to be used to attenuate blood loss and its associated risks in the postoperative period in this vulnerable patient population.

## Supplementary Information

Supplementary Material 1. Search strategies. MEDLINE (Ovid). EMBASE (Ovid). Web of Science Core Collection. Google Scholar (advanced search). Table S1: Grey literature. Table S2: Individual study characteristics. Table S3: Study characteristics – summarised. Table S4: Patient and intervention characteristics – details of individual studies. Table S5: Patient and intervention characteristics – summarised. Table S6: Findings of studies with a primary aim related to TXA. Table S7: Summary of findings for studies with a primary aim related to TXA. Table S8: Findings of studies with a primary aim not related to TXA. Table S9: Summary of findings for studies with a primary aim not related to TXA. Table S10: Findings of grey literature search. Figure S1: Publications on TXA in children with cerebral palsy undergoing hip or spine surgery.

Supplementary Material 2.

## Data Availability

All data extracted from included studies and used to synthesise findings in this review are included in the Supplementary Material.
