# Supplementary material for: Tranexamic acid in hip and spine surgery for children with cerebral palsy — a PRISMA-compliant scoping review
Source: Syst Rev. 2024 Dec 27;13:315. doi: 10.1186/s13643-024-02734-7 (PMC11673357; doi:10.1186/s13643-024-02734-7)

Contents

[Search strategies 2](#_Toc172950661)

[MEDLINE (Ovid) 2](#_Toc172950662)

[EMBASE (Ovid) 3](#_Toc172950663)

[Web of Science Core Collection 4](#_Toc172950664)

[Google Scholar (advanced search) 5](#_Toc172950665)

[Table S1: Grey literature 6](#_Toc172950666)

[Completed data charting forms 8](#_Toc172950667)

[Table S2: Individual study characteristics 8](#_Toc172950668)

[Table S3: Study characteristics – summarised 25](#_Toc172950669)

[Table S4: Patient and intervention characteristics – details of individual studies 27](#_Toc172950670)

[Table S5: Patient and intervention characteristics – summarised 44](#_Toc172950671)

[Table S6: Findings of studies with a primary aim related to TXA 45](#_Toc172950672)

[Table S7: Summary of findings for studies with a primary aim related to TXA 70](#_Toc172950673)

[Table S8: Findings of studies with a primary aim not related to TXA 74](#_Toc172950674)

[Table S9: Summary of findings for studies with a primary aim not related to TXA 89](#_Toc172950675)

[Table S10: Findings of grey literature search 92](#_Toc172950676)

# Search strategies

## MEDLINE (Ovid)

- <https://access.ovid.com/custom/redirector/index.html?dest=https://go.openathens.net/redirector/unimelb.edu.au?url=http://ovidsp.ovid.com/ovidweb.cgi?T=JS&CSC=Y&NEWS=N&PAGE=titles&SEARCHNAME=TXA+scoping+review+MEDLINE+search+v2&SEARCHTYPE=ps&SEARCHLEVEL=pin&D=medall>
- 1. Tranexamic Acid.mp. [mp=title, book title, abstract, original title, name of substance word, subject heading word, floating sub-heading word, keyword heading word, organism supplementary concept word, protocol supplementary concept word, rare disease supplementary concept word, unique identifier, synonyms, population supplementary concept word, anatomy supplementary concept word]

2. Orthopedic*.mp. [mp=title, book title, abstract, original title, name of substance word, subject heading word, floating sub-heading word, keyword heading word, organism supplementary concept word, protocol supplementary concept word, rare disease supplementary concept word, unique identifier, synonyms, population supplementary concept word, anatomy supplementary concept word]

3. Orthopaedic*.mp. [mp=title, book title, abstract, original title, name of substance word, subject heading word, floating sub-heading word, keyword heading word, organism supplementary concept word, protocol supplementary concept word, rare disease supplementary concept word, unique identifier, synonyms, population supplementary concept word, anatomy supplementary concept word]

4. (pediat* OR paediat* OR child* OR adolesc* OR bone* OR bony OR joint* OR acetab* OR ACL OR PCL OR ligament* OR reconstruct* OR re-construct* OR ankle* OR replace* OR bankart OR repair* OR shoulder* OR cartilag* OR spine* OR spinal* OR deform* OR disk* OR vertebral disk OR vertebral disks OR vertebral disc OR vertebral discs OR diskectomy OR discectomy OR radius OR radial OR radioulna* OR radio-ulna* OR ulna* OR arthro* OR elbow* OR fix* OR fusion OR fuse* OR fusing OR foot OR osteo* OR rod OR hemipelvec* OR hemiverteb* OR decompress* OR de-compress* OR revise OR revised OR revision OR revize OR revized OR re-do surgery OR re-do procedure OR re-do operation OR reoperation OR re-operation OR laminec* OR lengthen* OR salvag* OR mehta OR scolio* OR menisc* OR multiligament* OR multi-ligament OR delayed union OR malunion OR mal-union OR nonunion OR non-union OR knee OR pelvi* OR periacetab* OR peri-acetab* OR physeal* OR cruciate OR trauma OR rotationplast* OR sacrectom* OR sacro* OR sacrum OR cocc* OR tendo* OR teno* OR tommy john OR verteb* OR wrist OR fractur* OR spondyl* OR malform* OR injur* OR synov* OR arthrit* OR disloc* OR sublu* OR fibular deficiency OR pseudoarthrosis OR pseudo-arthrosis OR tibia* OR diaphysis OR metaphysis OR epiphysis OR clubfoot OR club-foot OR flatfoot OR flat-foot OR pes planus OR hallux OR brachymetatarsia OR kohler* OR apophysitis OR syndesmo* OR sprengel* OR scapula* OR clavic* OR olecranon OR fibrosarcoma OR rhabdomyosarcoma OR humerus OR humeral OR glenoid OR glenohumeral OR gleno-humeral OR disc* OR kypho* OR lordo* OR hip OR femor* OR femur OR disarticulation OR dis-articulation OR intraarticular OR intra-articular OR impingement OR calcan* OR talus OR talar OR forefoot OR fore-foot OR hindfoot OR hind-foot OR midfoot OR mid-foot OR equinus OR bone graft OR masquelet OR ilizarov OR coronoid OR fasci* OR acromio* OR congenital* OR debrid* OR sterno* OR styloid* OR scapho* OR carp* OR carpal OR tars* OR tarsal OR tarsus OR interphalang* OR inter-phalang* OR metaphalang* OR meta-phalang* OR intertars* OR inter-tarsal OR metatars* OR meta-tarsal OR volar OR washout OR wash-out OR Galeazzi OR Monteggia OR nail* OR intramedul* OR intra-medul* OR spica OR capsul* OR patell* OR malleol* OR diastasis OR tenoto* OR trochant* OR trochlea OR stabil* OR articul* OR open reduc* OR kirschner OR k-wir* OR compartment syndrome OR phalan* OR epiphys* OR hemiepiphys* OR hemi-epiphys* OR coraco* OR metalware OR metal-ware OR tuberos* OR prosthe* OR labrum OR labral OR ossif* OR contractur* OR bursa* OR hamate OR fibrocartilag* OR fibro-cartilag* OR femoroacetab* OR femoro-acetab* OR ganglion OR ganglia OR exostect* OR talo* OR adduc* OR abduct* OR cerebral palsy OR neuromuscular OR neuro-muscular OR SEML* OR single event multilevel OR single-event multilevel OR single-event multi-level OR single event multi-level OR varus derotation osteotomy OR varus derotational osteotomy OR varus de-rotation osteotomy OR varus de-rotational osteotomy OR vdro OR subtal* OR sub-tal* OR os calcis OR condyl* OR supracondyl* OR supra-condylar OR Dwyer OR Zielke OR bone met* OR interspin* OR inter-spin* OR pedic* OR lamin* OR sublam* OR sub-lam* OR pars interart* OR pars inter-art* OR screw* OR colum* OR odont* OR lumba* OR lumbosacral OR lumbo-sacral).mp. [mp=title, book title, abstract, original title, name of substance word, subject heading word, floating sub-heading word, keyword heading word, organism supplementary concept word, protocol supplementary concept word, rare disease supplementary concept word, unique identifier, synonyms, population supplementary concept word, anatomy supplementary concept word]

5. 2 or 3 or 4

6. 1 and 5

## EMBASE (Ovid)

- <https://access.ovid.com/custom/redirector/index.html?dest=https://go.openathens.net/redirector/unimelb.edu.au?url=http://ovidsp.ovid.com/ovidweb.cgi?T=JS&CSC=Y&NEWS=N&PAGE=titles&SEARCHNAME=TXA+scoping+review+EMBASE&SEARCHTYPE=ps&SEARCHLEVEL=pin&D=emcz>
- 1. exp tranexamic acid/

2. (orthopedic* or orthopaedic*).mp. [mp=title, abstract, heading word, drug trade name, original title, device manufacturer, drug manufacturer, device trade name, keyword heading word, floating subheading word, candidate term word]
3. (pediat* or paediat* or child* or adolesc* or bone* or bony or joint* or acetab* or ACL or PCL or ligament* or reconstruct* or re-construct* or ankle* or replace* or bankart or repair* or shoulder* or cartilag* or spine* or spinal* or deform* or disk* or vertebral disk or vertebral disks or vertebral disc or vertebral discs or diskectomy or discectomy or radius or radial or radioulna* or radio-ulna* or ulna* or arthro* or elbow* or fix* or fusion or fuse* or fusing or foot or osteo* or rod or hemipelvec* or hemiverteb* or decompress* or de-compress* or revise or revised or revision or revize or revized or re-do surgery or re-do procedure or re-do operation or reoperation or re-operation or laminec* or lengthen* or salvag* or mehta or scolio* or menisc* or multiligament* or multi-ligament or delayed union or malunion or mal-union or nonunion or non-union or knee or pelvi* or periacetab* or peri-acetab* or physeal* or cruciate or trauma or rotationplast* or sacrectom* or sacro* or sacrum or cocc* or tendo* or teno* or tommy john or verteb* or wrist or fractur* or spondyl* or malform* or injur* or synov* or arthrit* or disloc* or sublu* or fibular deficiency or pseudoarthrosis or pseudo-arthrosis or tibia* or diaphysis or metaphysis or epiphysis or clubfoot or club-foot or flatfoot or flat-foot or pes planus or hallux or brachymetatarsia or kohler* or apophysitis or syndesmo* or sprengel* or scapula* or clavic* or olecranon or fibrosarcoma or rhabdomyosarcoma or humerus or humeral or glenoid or glenohumeral or gleno-humeral or disc* or kypho* or lordo* or hip or femor* or femur or disarticulation or dis-articulation or intraarticular or intra-articular or impingement or calcan* or talus or talar or forefoot or fore-foot or hindfoot or hind-foot or midfoot or mid-foot or equinus or bone graft or masquelet or ilizarov or coronoid or fasci* or acromio* or congenital* or debrid* or sterno* or styloid* or scapho* or carp* or carpal or tars* or tarsal or tarsus or interphalang* or inter-phalang* or metaphalang* or meta-phalang* or intertars* or inter-tarsal or metatars* or meta-tarsal or volar or washout or wash-out or Galeazzi or Monteggia or nail* or intramedul* or intra-medul* or spica or capsul* or patell* or malleol* or diastasis or tenoto* or trochant* or trochlea or stabil* or articul* or open reduc* or kirschner or k-wir* or compartment syndrome or phalan* or epiphys* or hemiepiphys* or hemi-epiphys* or coraco* or metalware or metal-ware or tuberos* or prosthe* or labrum or labral or ossif* or contractur* or bursa* or hamate or fibrocartilag* or fibro-cartilag* or femoroacetab* or femoro-acetab* or ganglion or ganglia or exostect* or talo* or adduc* or abduct* or cerebral palsy or neuromuscular or neuro-muscular or SEML* or single event multilevel or single-event multilevel or single-event multi-level or single event multi-level or varus derotation osteotomy or varus derotational osteotomy or varus de-rotation osteotomy or varus de-rotational osteotomy or vdro or subtal* or sub-tal* or os calcis or condyl* or supracondyl* or supra-condylar or Dwyer or Zielke or bone met* or interspin* or inter-spin* or pedic* or lamin* or sublam* or sub-lam* or pars interart* or pars inter-art* or screw* or colum* or odont* or lumba* or lumbosacral or lumbo-sacral).mp. [mp=title, abstract, heading word, drug trade name, original title, device manufacturer, drug manufacturer, device trade name, keyword heading word, floating subheading word, candidate term word]
4. 2 or 3
5. 1 and 4

## Web of Science Core Collection

- ALL=((tranexamic acid OR TXA)))
  AND
  AK=((orthopedic OR orthopaedic OR orthopedics OR orthopaedics OR pediat* OR paediat* OR child* OR adolesc* OR bone* OR bony OR joint* OR acetab* OR ACL OR PCL OR ligament* OR reconstruct* OR re-construct* OR ankle* OR replace* OR bankart OR repair* OR shoulder* OR cartilag* OR spine* OR spinal* OR deform* OR disk* OR vertebral disk OR vertebral disks OR vertebral disc OR vertebral discs OR diskectomy OR discectomy OR radius OR radial OR radioulna* OR radio-ulna* OR ulna* OR arthro* OR elbow* OR fix* OR fusion OR fuse* OR fusing OR foot OR osteo* OR rod OR hemipelvec* OR hemiverteb* OR decompress* OR de-compress* OR revise OR revised OR revision OR revize OR revized OR re-do surgery OR re-do procedure OR re-do operation OR reoperation OR re-operation OR laminec* OR lengthen* OR salvag* OR mehta OR scolio* OR menisc* OR multiligament* OR multi-ligament OR delayed union OR malunion OR mal-union OR nonunion OR non-union OR knee OR pelvi* OR periacetab* OR peri-acetab* OR physeal* OR cruciate OR trauma OR rotationplast* OR sacrectom* OR sacro* OR sacrum OR cocc* OR tendo* OR teno* OR tommy john OR verteb* OR wrist OR fractur* OR spondyl* OR malform* OR injur* OR synov* OR arthrit* OR disloc* OR sublu* OR fibular deficiency OR pseudoarthrosis OR pseudo-arthrosis OR tibia* OR diaphysis OR metaphysis OR epiphysis OR clubfoot OR club-foot OR flatfoot OR flat-foot OR pes planus OR hallux OR brachymetatarsia OR kohler* OR apophysitis OR syndesmo* OR sprengel* OR scapula* OR clavic* OR olecranon OR fibrosarcoma OR rhabdomyosarcoma OR humerus OR humeral OR glenoid OR glenohumeral OR gleno-humeral OR disc* OR kypho* OR lordo* OR hip OR femor* OR femur OR disarticulation OR dis-articulation OR intraarticular OR intra-articular OR impingement OR calcan* OR talus OR talar OR forefoot OR fore-foot OR hindfoot OR hind-foot OR midfoot OR mid-foot OR equinus OR bone graft OR masquelet OR ilizarov OR coronoid OR fasci* OR acromio* OR congenital* OR debrid* OR sterno* OR styloid* OR scapho* OR carp* OR carpal OR tars* OR tarsal OR tarsus OR interphalang* OR inter-phalang* OR metaphalang* OR meta-phalang* OR intertars* OR inter-tarsal OR metatars* OR meta-tarsal OR volar OR washout OR wash-out OR Galeazzi OR Monteggia OR nail* OR intramedul* OR intra-medul* OR spica OR capsul* OR patell* OR malleol* OR diastasis OR tenoto* OR trochant* OR trochlea OR stabil* OR articul* OR open reduc* OR kirschner OR k-wir* OR compartment syndrome OR phalan* OR epiphys* OR hemiepiphys* OR hemi-epiphys* OR coraco* OR metalware OR metal-ware OR tuberos* OR prosthe* OR labrum OR labral OR ossif* OR contractur* OR bursa* OR hamate OR fibrocartilag* OR fibro-cartilag* OR femoroacetab* OR femoro-acetab* OR ganglion OR ganglia OR exostect* OR talo* OR adduc* OR abduct* OR cerebral palsy OR neuromuscular OR neuro-muscular OR SEML* OR single event multilevel OR single-event multilevel OR single-event multi-level OR single event multi-level OR varus derotation osteotomy OR varus derotational osteotomy OR varus de-rotation osteotomy OR varus de-rotational osteotomy OR vdro OR subtal* OR sub-tal* OR os calcis OR condyl* OR supracondyl* OR supra-condylar OR Dwyer OR Zielke OR bone met* OR interspin* OR inter-spin* OR pedic* OR lamin* OR sublam* OR sub-lam* OR pars interart* OR pars inter-art* OR screw* OR colum* OR odont* OR lumba* OR lumbosacral OR lumbo-sacral)

NB: I selected 'author keywords' because there was a 50-term limit on other fields, i.e. I could not include all of these search terms in an 'ALL' search

## Google Scholar (advanced search)

- **(with all of the words)** tranexamic acid OR TXA
- **(with at least one of the words)** orthopaedic OR orthopedic OR pediatric OR paediatric OR child OR adolescent OR bone OR bony OR joint OR acetabulum OR acetabular OR acl OR pcl OR ligament OR reconstruct OR ankle OR replace OR bankart OR repair OR shoulder OR cartilage OR cartilaginous OR spine OR spinal OR deform OR disk OR vertebral disk OR vertebral disc OR diskectomy OR discectomy OR disc OR radius OR radial OR radioulna OR radio-ulna OR ulna OR arthroscopy OR arthroplasty OR elbow OR fix OR fusion OR fuse OR fusing OR foot OR osteo OR rod OR hemipelvectomy OR hemivertebra OR decompression OR de-compression OR revise OR revize OR re-do surgery OR re-do procedure OR re-operation OR reoperation OR revision OR laminectomy OR lengthen OR salvage OR salvaging OR mehta OR scoliosis OR meniscus OR meniscal OR meniscectomy OR multiligament OR multi-ligament OR delayed union OR malunion OR mal-union OR nonunion OR non-union OR knee OR pelvis OR pelvic OR periacetabular OR peri-acetabular OR physeal OR cruciate OR trauma OR rotationplasty OR sacrectomy OR sacrum OR sacral OR sacroiliac OR sacro-iliac OR coccyx OR tendon OR tenodesis OR tommy john OR collateral OR vertebra OR wrist OR fracture OR spondylosis OR malformation OR malformed OR injury OR injured OR synovium OR synovial OR synovectomy OR arthritis OR arthritic OR dislocation OR dislocated OR subluxation OR subluxed OR fibular OR fibula OR arthrosis OR pseudoarthrosis OR pseudo-arthrosis OR tibia OR diaphysis OR metaphysis OR epiphysis OR clubfoot OR club-foot OR flatfoot OR flat-foot OR pes planus OR hallux OR brachymetatarsia OR kohler OR apophysitis OR syndesmosis OR syndesmotic OR sprengel OR scapula OR clavicle OR clavicular OR olecranon OR fibrosarcoma OR rhabdomyosarcoma OR humerus OR humeral OR glenoid OR glenohumeral OR gleno-humeral OR kyphosis OR kyphotic OR lordosis OR lordotic OR hip OR femoral OR femur OR disarticulation OR dis-articulation OR intraarticular OR intra-articular OR impingement OR calcaneus OR calcaneal OR talus OR talar OR forefoot OR fore-foot OR hindfoot OR hind-foot OR midfoot OR mid-foot OR equinus OR bone graft OR masquelet OR ilizarov OR coronoid OR fascia OR fasciotomy OR fasciectomy OR acromion OR acromial OR acromioclavicular OR acromio-clavicular OR congenital OR debride OR debriding OR sternoclavicular OR sterno-clavicular OR styloid OR scaphoid OR carpus OR carpal OR tarsal OR tarsus OR interphalangeal OR inter-phalangeal OR metaphalangeal OR meta-phalangeal OR intertarsal OR inter-tarsal OR metatarsal OR meta-tarsal OR volar OR washout OR wash-out OR Galeazzi OR Monteggia OR nail OR intramedullary OR intra-medullary OR spica OR capsule OR capsulotomy OR patella OR patellofemoral OR patello-femoral OR malleolus OR malleoli OR diastasis OR tenotomy OR trochlea OR trochanter OR stability OR stabilize OR stabilise OR stabilizing OR stabilising OR articular OR reduce OR reduction OR kirschner OR k-wire OR k wire OR compartment syndrome OR phalanx OR phalanges OR phalangeal OR epiphysis OR hemiepiphysis OR hemi-epiphysis OR coracoacromial OR coraco-acromial OR metalware OR metal-ware OR plate OR tuberosity OR prosthesis OR prosthetic OR labrum OR labral OR ossification OR ossify OR contracture OR bursa OR hamate OR fibrocartilage OR fibro-cartilage OR arthrotomy OR femoroacetabular OR femoro-acetabular OR ganglion OR ganglia OR exostectomy OR talocalcaneal OR talo-calcaneal OR adductor OR abductor OR cerebral palsy OR neuromuscular OR neuro-muscular OR semls OR seml OR single event multilevel surgery OR single-event multilevel surgery OR single-event multi-level surgery OR single event multi-level surgery OR varus derotation osteotomy OR varus derotational osteotomy OR varus de-rotation osteotomy OR varus de-rotational osteotomy OR vdro OR subtalar OR sub-talar OR os calcis OR condylar OR supracondylar OR supra-condylar OR Dwyer OR Zielke OR bone OR metastasis OR bone metastases OR bone mets OR interspinous OR inter-spinous OR pedicle OR laminectomy OR sublaminar OR sub-laminar OR pars interarticularis OR pars inter-articularis OR screw OR column OR odontoid OR lumbar OR lumbosacral OR lumbo-sacral

## Table S1: Grey literature

| **Source** | **Terms/search strategy in this platform/source** |
| --- | --- |
| WHO International Clinical Trials Registry Platform (ICTRP) | Tranexamic acid   - Filtered search by ‘clinical trials in children’ |
| clinaltrials.gov | Tranexamic acid   - Filtered search by ‘Child’ age range |
| New York Academy of Medicine Grey Literature Report | Using Advanced Search restricted to tick box for Grey Literature, ‘tranexamic acid’ returned no results. ‘antifibrinolytic’ returned 8 results |
| Open Science Framework | Tranexamic acid   - Filtered search by ‘Registrations’ |
| ProQuest | Tranexamic acid   - Filtered to subject = ‘surgery’ |
| Dimensions | ‘Tranexamic acid’ - no filters.  Screened records from the following sections: Datasets, Grants, and Policy Documents. |
| Trove | ‘Tranexamic acid – no filters |
| WHO IRIS | Tranexamic acid – no filters |
| General Google search | Each term under the ‘tranexamic acid’ scope note in Medline was searched in Google:   - 1197-18-8 (tranexamic acid) - 6t84r30kc1 - amca - amcha - amchafibrin - anvitoff - cyklokapron - exacyl - kabi 2161 - spotof - tranexamic acid - transamin - ugurol - t-amcha - trans-4-(aminomethyl)cyclohexanecarboxylic acid |

# Completed data charting forms

## Table S2: Individual study characteristics

| **Record ID (first author, year of publication)** | **Type of record and source** | ***Type of study** | **Setting and study period** | **Patient population and surgical procedure** | ****Primary aim related to TXA** |
| --- | --- | --- | --- | --- | --- |
| Bekmez et al. 2018  DOI: 10.1097/BRS.0000000000002538 | Journal article  Source: primary literature search | Case series | Single centre, Turkey, study period not reported | Population: neuromuscular scoliosis patients  Sample size: 22  Proportion with CP: 9/22 = 40.9%  Surgical procedure: pedicle subtraction osteotomy group or posterior column osteotomy | No |
| Adler et al. 2022  DOI: 10.1111/vox.13372. | Journal article  Source: primary literature search | Cohort study (retrospective) | Single centre, USA, January 2012 to April 2021 | Population: hip dysplasia patients  Sample size: 565  Proportion with CP: unclear (268/565 = 47.4% with neuromuscular dysplasia, which includes CP)  Surgical procedure: periacetabular osteotomy +/- femoral osteotomy | Not directly (TXA was analysed as one of the factors potentially associated with the need for allogeneic blood transfusion) |
| Sethna et al. 2005  DOI: 10.1097/00000542-200504000-00006 | Journal article  Source: primary literature search | RCT (no indication of prospective trial registration) | Single centre, USA, study period not reported | Population: scoliosis (idiopathic or secondary)  Sample size: 44  Proportion with CP: 1/44 = 2.3%  Surgical procedure: scoliosis correction surgery with posterior spinal instrumentation (although some patients underwent some sort of antero-posterior surgery) | Yes |
| Brouwer et al. 2021:  DOI: 10.1302/1863-2548.15.200249 | Journal article  Source: primary literature search | Cohort study (retrospective) | Single centre, Netherlands, 1 Jan 2014 to 31 July 2019 | Population: hip dysplasia  Sample size: 263 patients (340 procedures)  Proportion with CP: ***123 procedures for secondary dysplasia including CP = 123/340 = 36.2%  Surgical procedure: proximal femoral osteotomy (n = 117 (34.4%)), pelvic osteotomy (n =99 (29.1%)), proximal femoral and pelvic osteotomy (n = 124 (36.5%)) | Yes |
| Lu et al. 2020  DOI: 10.1111/ans.16288 | Journal article  Source: primary literature search | Cohort study (retrospective) | Single centre, Australia, 2010 to 2015 | Population: CP patients  Sample size: 134  Proportion with CP: 100%  Surgical procedure: mixed spine/hip surgery cohort (98 SEMLS, 36 spinal surgery) | Not directly (primary aim of this study was to assess factors associated with allogeneic blood transfusion in patients with CP undergoing major orthopaedic surgery, and TXA was one of these factors) |
| Compton et al. 2022  DOI: 10.1097/MD.0000000000028506 | Journal article  Source: primary literature search | Cohort study (retrospective) | Single centre, USA, January 2, 2004 to August 22, 2019 | Population: CP patients  Sample size: 390  Proportion with CP: 100%  Surgical procedure: VDRO | Yes |
| McLeod et al. 2015  DOI: 10.1097/BSD.0b013e3182a22a54 | Journal article  Source: primary literature search | Cohort study (retrospective) | Multicentre, USA, January 1, 2006 to September 30, 2009 | Population: paediatric patients with scoliosis  Sample size: 4269  Proportion with CP: 536/4269 = 12.6%  Surgical procedure: corrective spinal surgery | Not directly ('antifibrinolytics', not specifically TXA) |
| Tzatzairis et al. 2020  DOI: 10.1007/s00590-020-02663-w | Journal article  Source: primary literature search | Cohort study (retrospective) | Single centre, UK, May 2012 to January 2019 | Population: CP patients <16 years of age  Sample size: 51  Proportion with CP: 100%  Surgical procedure: unilateral or bilateral VDRO with soft tissue release or bilateral VDRO with soft tissue release without pelvic osteotomy | Yes |
| McNeil et al. 2020  DOI: 10.1097/BRS.0000000000003273 | Journal article  Source: primary literature search | Cohort study (retrospective) | Multicentre database, USA, 2016 to 2017 | Population: paediatric patients who underwent posterior multilevel spine surgery  Sample size: 604 after propensity score matching  Proportion with CP: 10/604 = 1.7%  Surgical procedure: elective posterior multilevel spine surgery,  seven or more vertebral levels: 10 patients with CP | Yes |
| Nazareth et al. 2018  DOI: 10.1302/1863-2548.13.180143 | Journal article  Source: primary literature search | Cohort study (retrospective) | Single centre, USA, 1 November  2004 to 2 February 2017 | Population: CP patients  Sample size: 258  Proportion with CP: 100%  Surgical procedure: VDRO | Yes |
| Abbot et al. 2014  DOI: 10.1097/BRS.0000000000000466 | Journal article  Source: primary literature search | Cohort study (retrospective) | Single centre, USA, June 2007 to August 2011 | Population: paediatric patients who underwent a posterior spinal deformity correction surgery  Sample size: 117  Proportion with CP: not reported, but 41/117 = 35.0% with neuromuscular scoliosis  Surgical procedure: posterior spinal deformity correction surgery | No |
| Bichmann et al. 2023  DOI: 10.1177/21925682231166109 | Conference abstract  Source: primary literature search | Cohort study (retrospective) | Single centre, Germany, November 2014 to December 2020 | Population: Patients with non-idiopathic scoliosis  Sample size: 66  Proportion with CP: not reported  Surgical procedure: primary posterior spinal fusion | Yes |
| Bird et al. 2011  DOI: 10.1111/j.1460-9592.2010.03443.x | Journal article  Source: primary literature search | Survey | Multicentre, UK, study period not reported | Population: anaesthetists from UK hospitals at which paediatric and adolescent scoliosis surgery  Sample size: At least one anaesthetist from 21 of 24 hospitals  Proportion with CP: not applicable  Surgical procedure: scoliosis correction surgery | Not directly (the impact of blood conservation strategies was an aim, and TXA was suggested as part of these strategies) |
| Chiem et al. 2017  DOI: 10.1111/pan.13141 | Journal article  Source: primary literature search | Case report | Single centre, USA, study period not reported | Population: patient with neuromuscular scoliosis  Sample size: 1 (case report)  Proportion with CP: unclear (aetiology not specified)  Surgical procedure: posterior spinal fusion | No |
| Colomina et al. 2009  DOI: 10.3928/01477447-20090201-12 | Journal article  Source: primary literature search | Case control | Single centre, Spain, 2001 to 2003 | Population: patients undergoing complex spine surgery  Sample size: 69  Proportion with CP: not specified but 15/69 = 21.7% with neuromuscular scoliosis  Surgical procedure: complex spine surgery | Yes |
| Degiorgio-Miller et al. 2013  DOI: 10.1111/dmcn.12247 | Conference abstract  Source: primary literature search | Unclear (proportion of patients in whom TXA was used was not reported) | Single centre, New Zealand, 1992 to 2006 | Population: CP patients with spastic quadriplegia  Sample size: 28  Proportion with CP: 100%  Surgical procedure: If pelvic obliquity was greater than 20 degrees, fusion was performed to the sacrum. If less than 20 degrees, fusion was performed to the lumbar spine, sparing the lumbosacral junction thereby preserving mobility | No |
| Dhawale et al. 2012  DOI: 10.1097/BRS.0b013e31823d009b | Journal article  Source: primary literature search | Cohort study (retrospective) | Multicentre, USA, January 2008 to December 2010 | Population: CP patients with scoliosis  Sample size: 84  Proportion with CP: 100%  Surgical procedure: posterior spinal fusion | Not directly ('antifibrinolytic', not specifically TXA) |
| Dick et al. 2019  DOI: 10.1177/2192568219837488 | Journal article  Source: primary literature search | Case series | Single centre, UK, January 2001 to December 2015 | Population: paediatric patients with scoliosis  Sample size: 1039  Proportion with CP: 72/1039 = 6.9%  Surgical procedure: scoliosis surgery (not further specified) | Not directly (the aim of the study was to evaluate the impact of a multifaceted, multidisciplinary blood conservation integrated care pathway program - TXA was part of this pathway) |
| Dong et al. 2021  DOI: 10.1186/s12891-020-03869-z | Journal article  Source: primary literature search | Case series | Single centre, China, January 2014 to December 2017 | Population: adolescent patients with scoliosis  Sample size: 722  Proportion with CP: not specified but 42/722 = 5.8% with neuromuscular scoliosis  Surgical procedure: scoliosis surgery (not further specified) | Not directly (the aim was to investigate risk factors for blood transfusion, and TXA was one of the factors investigated) |
| Dowlut et al. 2017  DOI: not available | Conference abstract  Source: primary literature search | Cohort study (retrospective) | Single centre, UK, June 2014 to July 2015 | Population: paediatric patients undergoing hip reconstructive surgery  Sample size: 33  Proportion with CP: not reported  Surgical procedure: hip reconstruction (not further specified) | Yes |
| Dupuis et al. 2015  DOI: 10.1016/j.accpm.2015.04.003 | Journal article  Source: primary literature search | Case series | Single centre, France, January 2012 to December 2012 | Population: paediatric scoliosis patients  Sample size: 147  Proportion with CP: 9/147 = 6.1%  Surgical procedure: scoliosis surgery, including but not limited to fusion (not further specified) | Not directly (the aim was to investigate risk factors for blood transfusion, and TXA was one of the factors investigated) |
| Duvernay et al. 2020  DOI: 10.1182/blood-2020-138730 | Journal article  Source: primary literature search | Case series | Single centre, USA, study period not reported | Population: paediatric scoliosis patients  Sample size: 8  Proportion with CP: not specified  Surgical procedure: scoliosis surgery (not further specified) | No |
| Eisler et al. 2020  DOI: 10.1097/BRS.0000000000003455. | Journal article  Source: primary literature search | Cohort study (retrospective) | Multicentre database, USA, 2016 to 2017 | Population: paediatric scoliosis patients  Sample size: 7982 patients, 6626 of these included in the complete case analysis, including 1192 propensity score matched pairs  Proportion with CP: 830/7982 = 10.4%  Surgical procedure: spinal fusion | Not directly ('antifibrinolytic', not specifically TXA) |
| Ezhevskaya et al. 2015  DOI: not available | Conference abstract  Source: primary literature search | RCT (no indication of prospective trial registration) | Single centre, Russia, study period not reported | Population: patients with scoliosis aged 15 to 18 years  Sample size: 115  Proportion with CP: not specified  Surgical procedure: scoliosis surgery (not further specified) | Yes |
| Fernandez et al. 2021  DOI: not available | Conference abstract  Source: primary literature search | Case series | Single centre, Spain, study period not reported | Population: paediatric scoliosis patients  Sample size: 31  Proportion with CP: not specified but 32.3% had non-idiopathic scoliosis  Surgical procedure: scoliosis surgery (not further specified) | Not directly |
| Gibson et al. 2022  DOI: 10.1007/s43390-022-00489-6 | Journal article  Source: primary literature search | Case series | Single centre, USA, December 2019 to March 2021 | Population: paediatric patients with scoliosis  Sample size: 17  Proportion with CP: not specified but 9/17 = 52.3% with neuromuscular scoliosis  Surgical procedure: posterior spinal fusion | Yes |
| Gurajala et al. 2013  DOI: 10.4103/0019-5049.108554 | Journal article  Source: primary literature search | Cohort study (retrospective) | Single centre, India, January 2006 to July 2011 | Population: paediatric patients with scoliosis  Sample size: 102  Proportion with CP: not specified but 15/102 = 14.7% with neuroparalytic scoliosis  Surgical procedure: spinal fusion of the thoracolumbar region | Not directly (TXA was one of the factors analysed to determine whether it was associated with early extubation) |
| Halanski et al. 2014  DOI: 10.1016/j.jspd.2014.02.001 | Journal article  Source: primary literature search | Cohort study (retrospective) | Single centre, USA, January 2009 to  December 2010 | Population: paediatric patients with scoliosis  Sample size: 47  Proportion with CP: not specified but 10/47 = 21.3% with neuromuscular scoliosis  Surgical procedure: posterior spinal fusion | Yes |
| Ivasyk et al. 2022  DOI: 10.1186/s12891-022-05604-2 | Journal article  Source: primary literature search | Cohort study (retrospective) | Multicentre, USA, January 2013 to  December 2015 | Population: paediatric patients undergoing spinal fusion surgery  Sample size: 2633  Proportion with CP: not specified but reported >5% of patients had neuromuscular scoliosis  Surgical procedure: spinal fusion | Yes |
| Jurgens et al. 2017  DOI: 10.1111/anae.14060 | Conference abstract  Source: primary literature search | Case series | Single centre, UK, study period not reported | Population: scoliosis patients  Sample size: 51  Proportion with CP: not specified but 20/51 = 39.2% in the ‘neuromuscular and other’ group  Surgical procedure: scoliosis operation (not further specified) | Unclear |
| Koraki et al. 2020  DOI: 10.1007/s00590-020-02637-y | Journal article  Source: primary literature search | Unclear (proportion of patients in whom TXA was used was not reported) | Single centre, Greece, October  2011 to November 2016 | Population: adolescent scoliosis patients  Sample size: 35  Proportion with CP: not specified but no limitation reported on aetiology of scoliosis  Surgical procedure: posterior spinal arthrodesis | Not directly (TXA was part of a protocol evaluated for its impact on reducing allogeneic blood transfusion) |
| Lins et al. 2020  DOI: 10.1097/BPO.0000000000001534 | Journal article  Source: primary literature search | Cohort study (retrospective) | Single centre, USA, 2013 to 2018 | Population: patients with neuromuscular complex chronic conditions  Sample size: 166 total, of whom 119 were propensity score matched  Proportion with CP: not specified  Surgical procedure: hip reconstructive surgery | Yes |
| ****Lins et al. 2019  DOI: 10.1111/dmcn.14353 |  |  |  |  |  |
| Majid et al. 2015  DOI: 10.1155/2015/827027 | Journal article  Source: primary literature search | Cohort study (retrospective) | Single centre, UK, 2011 to 2013 | Population: children with cerebral palsy  Sample size: 51  Proportion with CP: 100%  Surgical procedure: hip reconstructive surgery. Non-TXA group = 10/37 (27%) bilateral procedure; 11/37 (29.7%) femoral osteotomy, 1/37 (2.7%) pelvic osteotomy only, 25/37 (67.6%) both femoral and pelvic osteotomy; TXA group = 8/14 (57%) bilateral; 3/14 (21.4%) femoral, 0 pelvic, 11/14 (78.6%) both | Yes |
| Masrouha et al. 2022  DOI: 10.1007/s00590-020-02858-1 | Journal article  Source: primary literature search | Cohort study (retrospective) | Single centre, USA, January 2013 to April 2019 | Population: children with cerebral palsy undergoing hip reconstruction with two or more osteotomies  Sample size: 43  Proportion with CP: 100%  Surgical procedure: 39 patients underwent two osteotomies (22 in  the TXA group and 17 in the non-TXA group) - either bilateral femoral osteotomies or unilateral  femoral and acetabular osteotomies. Two patients in each  group underwent three osteotomies (bilateral femoral osteotomies and a unilateral acetabular osteotomy) | Yes |
| *****Michelet et al. 2018  DOI: 10.1016/j.accpm.2017.03.003 |  |  |  |  |  |
| Mihas et al. 2021  DOI: 10.1186/s12891-021-04081-3 | Journal article  Source: primary literature search | Case series | Single centre, USA, 2014 to 2018 | Population: children with scoliosis who refused blood transfusion  Sample size: 20  Proportion with CP: not specified but 3/20 = 15% with neuromuscular scoliosis  Surgical procedure: spinal deformity surgery (not further specified) | Not directly (TXA was one part of the strategy evaluated to reduce blood loss) |
| Neilipovitz et al. 2001  DOI: 10.1097/00000539-200107000-00018 | Journal article  Source: primary literature search | RCT (no indication of prospective trial registration) | Single centre, Canada, study period not reported | Population: children with scoliosis  Sample size: 40  Proportion with CP: not specified but 25/40 = 62.5% with secondary scoliosis  Surgical procedure: posterior spinal fusion | Yes |
| Nellis et al. 2021  DOI: 10.1111/trf.16623 | Conference abstract  Source: primary literature search | Cohort study (retrospective) | Multicentre, USA, 2013 to 2016 | Population: children undergoing spinal fusion surgery  Sample size: 411  Proportion with CP: not specified but no reported limitation on inclusion of scoliosis in CP patients  Surgical procedure: spinal fusion surgery | Unclear |
| O'Donoghue et al. 2020  DOI: 10.1111/anae.14953 | Conference abstract  Source: primary literature search | Case series | Single centre, UK, study period not reported | Population: patients undergoing major spinal deformity corrective surgery  Sample size: 48  Proportion with CP: not specified but 7/48 = 14.6% with neuromuscular scoliosis  Surgical procedure: major spinal deformity correction surgery | Not directly (TXA was part of the strategy to reduce use of haematological resources) |
| Schur et al. 2017  DOI: 10.1097/BCO.0000000000000525 | Journal article  Source: primary literature search | Cohort study (retrospective) | Single centre, USA, June 2004 to March 2012 | Population: paediatric scoliosis patients  Sample size: 594  Proportion with CP: not specified, but 243/594 = 40.9% with neuromuscular scoliosis  Surgical procedure: posterior spinal fusion with minimum 10 levels fused | Yes |
| ******Schur et al. 2018  DOI: 10.1097/BPO.0000000000000851 | Journal article  Source: primary literature search | Cohort study (retrospective) | Single centre, USA, June 2004 to March 2012 | Population: paediatric scoliosis patients  Sample size: 510  Proportion with CP: not specified, but 223/510 = 43.7% with neuromuscular scoliosis  Surgical procedure: posterior spinal fusion with minimum 10 levels fused | No |
| Shrader et al. 2018  DOI: 10.1016/j.jspd.2018.03.002 | Journal article  Source: primary literature search | Case series | Single centre, USA, study period = 2012 for the 25 in the patients two-surgeon group, 2008-2010 for the matched one-surgeon group | Population: patients with CP and progressive neuromuscular scoliosis  Sample size: 50  Proportion with CP: 100%  Surgical procedure: posterior spinal fusion | No |
| Singh et al. 2013  DOI: 10.4103/0019-5049.123334 | Journal article  Source: primary literature search | Case report | Single centre, India, 2013 | Population: child with cerebral palsy scoliosis  Sample size: 1 (case report)  Proportion with CP: 100%  Surgical procedure: scoliosis correction surgery (not further specified) | No |
| Soini et al. 2023  DOI: 10.2340/17453674.2023.11962 | Journal article  Source: primary literature search | Case series | Single centre, Finland, 2009 to 2020 | Population: scoliosis patients  Sample size: 180  Proportion with CP: 16/180 = 8.9%  Surgical procedure: spinal fusion surgery (not further specified) | No |
| Spiessberger et al. 2023  DOI: 10.1007/s00381-023-05834-2 | Journal article  Source: primary literature search | Cohort study (retrospective) | Multicentre, USA and Canada, January 2003 to December 2018 | Population: patients aged <18 years at index surgery undergoing posterior cervical fusion for spinal deformity  Sample size: 79  Proportion with CP: not specified but 7/79 = 8.9% with neuromuscular scoliosis  Surgical procedure: posterior cervical spine fusion | No |
| Tan et al. 2018  DOI: not available | Journal supplement  Source: primary literature search | Cohort study (retrospective) | Single centre, USA, study period not reported | Population: paediatric patients undergoing posterior spinal fusion surgery  Sample size: 795  Proportion with CP: not specified, and also not specified what proportion of patients were in the neuromuscular scoliosis group  Surgical procedure: posterior spinal fusion | Not directly ('antifibrinolytics' were part of the overall approach to reducing transfusion) |
| van Kouswijk et al. 2023  DOI: 10.1177/18632521231199518 | Journal article  Source: primary literature search | RCT protocol (prospectively registered with EU Clinical Trials Register) | Single centre, Netherlands, study period not reported | Population: participants aged from 1 to 18 years undergoing PFPOs (proximal femoral and/or pelvic osteotomies)  Sample size: 180  Proportion with CP: not specified, and no specific recruitment target for different indications for surgery  Surgical procedure: proximal femoral and/or pelvic osteotomies | Yes |
| Vasan et al. 2021  DOI: 10.1007/s00586-021-06798-0 | Journal article  Source: primary literature search | Case series | Single centre, India, September 2017 to August 2018 | Population: paediatric patients undergoing posterior spinal deformity surgery  Sample size: 75  Proportion with CP: not specified but 15/75 = 20% with neuromuscular scoliosis  Surgical procedure: corrective surgery for scoliosis and kyphosis involving vertebral fusion≥6 levels and expected blood loss (BL)≥750 ml | No |
| Verma et al. 2010  DOI: 10.1186/1471-2482-10-13 | Journal article  Source: primary literature search | RCT protocol (prospectively registered on clinicaltrials.gov) | Single centre, USA, study period not reported | Population: patients with neuromuscular scoliosis, adolescent idiopathic scoliosis, or adult spinal deformity  Sample size: aiming maximum number of 390 patients overall  Proportion with CP: not specified but aiming to recruit maximum 90/390 = 23.1% patients with neuromuscular scoliosis  Surgical procedure: corrective spinal surgery | Yes |
| Vrbica et al. 2023  DOI: 10.1136/bmjopen-2022-071547 | Journal article  Source: primary literature search | RCT pilot study protocol (prospectively registered on clinicaltrials.gov) | Single centre, Czech Republic, pilot study  started on 1 June 2022 and the estimated end of the study  was planned on 30 September 2023 | Population: paediatric scoliosis patients  Sample size: aiming to recruit 30 patients overall  Proportion with CP: not specified but no stated exclusion for patients with CP or neuromuscular scoliosis  Surgical procedure: scoliosis surgery (not further specified) | No |
| Weissman et al. 2020  DOI: 10.1007/s00586-020-06572-8 | Journal article  Source: primary literature search | Cohort study (retrospective) | Single centre, Chile, 2009 to 2016 | Population: patients <25 years old who underwent spinal deformity surgery for scoliosis  Sample size: 181  Proportion with CP: not specified but 88/181 = 48.6% with neuromuscular scoliosis  Surgical procedure: posterior spinal deformity surgery | Yes |
| Zuccon 2023  DOI: 10.3390/children10121931 | Journal article  Source: grey literature search | RCT (no indication of prospective registration) | Single centre, Brazil, 2018 to 2019 | Population: patients <18 years old with cerebral palsy  Sample size: 31  Proportion with CP: 100%  Surgical procedure: unilateral surgical hip reconstruction | Yes |
| Fernandes 2020  DOI: 10.1155/2020/8246309 | Journal article  Source: grey literature search | Cohort study (retrospective) | Single centre, Portugal, 2006 to 2016 | Population: patients <21 years old with scoliosis or kyphosis who underwent surgical correction  Sample size: 209  Proportion with CP: not specified by 100/209 = 47.8% with neuromuscular scoliosis  Surgical procedure: posterior spinal fusion | Not directly (TXA was part of a multimodal strategy to reduce blood loss) |
| *’type of study’ defined from the perspective of tranexamic acid (TXA) - e.g. if all patients in a study received TXA, it was considered a case series; **’not directly’ means the study aim incorporated TXA but TXA was not the whole study aim; ***number of patients with CP not reported; ****included in this table for completeness - this conference abstract presents the study that was later published as Lins 2020; RCT = randomised controlled trial; *****included in this table for completeness – this is the exact same study cohort as Dupuis et al. 2015 and the article provided no additional information relevant to this scoping review, therefore extracted data were not included; ******study population drawn from the same database as that used in Schur et al. 2017, but slightly different inclusion criteria and different focus of study therefore slightly different study population and findings thus both studies were included for analysis; CP = cerebral palsy; VDRO = varus derotation osteotomy; SEMLS = single-event multilevel surgery | | | | | |

## Table S3: Study characteristics – summarised

| **Characteristic** | **Findings** |
| --- | --- |
| Type of record (n (% of total)) | Journal article = 43 (82.7%)  Conference abstract = 8 (15.4%)  Journal supplement = 1 (1.9%) |
| Country in which study was conducted (n (% of total)) | *Country:   - USA = 24 (46.2%) - UK = 7 (13.5%) - India = 3 (5.8%) - Two (3.8%) from each of: Spain, Turkey, Netherlands - One (1.9%) from each of: Australia, Germany, New Zealand, China, Russia, Greece, Canada, Finland, Czech Republic, Chile, Brazil, Portugal, France |
| Study period | Range = 1992 to 2023  Mean (range) study duration in years = 5.0 (1 to 15)  Not reported (n (% of total)) = 13 (25.0%) |
| Surgical procedure | In spine studies (n (% of total)):   - Pedicle subtraction osteotomy group or posterior column osteotomy = 1 (2.5%) - Scoliosis correction surgery with posterior spinal instrumentation = 1 (2.5%) - Corrective spinal surgery (not further specified) = 5 (12.5%) - Posterior multilevel spine surgery with ≥7 vertebral levels = 1 (2.5%) - Posterior spinal deformity surgery = 2 (5.0%) - Posterior spinal fusion (two records specified minimum 10 levels fused) = 14 (35.0%) - Scoliosis surgery (not further specified) = 10 (25.0%) - Spinal fusion surgery (not limited to posterior fusion) = 5 (12.5%) - Corrective surgery for scoliosis and kyphosis involving vertebral fusion ≥6 levels and expected blood loss ≥750 mL = 1 (2.5%)   In hip studies (n (% of total)):   - Femoral +/- pelvic osteotomy, unilateral or bilateral = 5 (45.5%) - Unilateral hip reconstruction = 1 (9.1%) - VDRO = 3 (27.3%) - Hip reconstruction (not further specified) = 2 (18.2%)   In mixed hip/spine studies (n (% of total)):   - Spinal surgery or SEMLS = 1 (100%) |
| Primary study aim related to TXA (n (% of total)) | Yes = 22 (42.3%)  Not directly = 16 (30.8%)  No = 12 (23.1%)  Missing/unable to determine study aim = 2 (3.8%) |
| CP = cerebral palsy; VDRO = varus derotational osteotomy; SEMLS = single-event, multi-level surgery; *does not sum to 54 because multicentre studies can include multiple countries; **number with CP or neuromuscular condition; excluding the survey study but including the targeted sample size of RCT protocols; ***excluding survey study | |

## Table S4: Patient and intervention characteristics – details of individual studies

| **Record ID** | ***Age** | **Sex** | **Functional level** | **Tranexamic acid dosing** | **Primary outcome** | **Sample size calculation** |
| --- | --- | --- | --- | --- | --- | --- |
| **Spine studies (AND SPINE COMPONENT OF LU 2020)** | | | | | | |
| Bekmez et al. 2018 | Multiple posterior column osteotomies (PCOs) group = 15.6 years (range 13-21)  Apical pedicle subtraction osteotomy (PSO) group and: PCO group = 15.8 (11-40) | 11 (50%) female | Wheelchair-dependent | “Appropriate dose” not further specified | Unclear | Not carried out |
| Sethna et al. 2005 | TXA group = mean 13.6 years (SD 1.8)  Placebo group = 14.0 (2.0) | TXA group = 11 (64.7%) female  Non-TXA group = 8 (38.1%) female | Not reported | 100 mg/kg over 15 minutes after induction of anaesthesia and before skin incision, followed by 10mg/kg/hr infusion until skin closure. | Intraoperative blood loss | A priori = yes  Target sample size achieved = yes |
| Lu et al. 2020 | Spinal surgery group = mean 13.8 years (SD 2.0) | Spinal surgery group = 19 (52.8%) female | GMFCS 1 = 0  GMFCS 2 = 1 (2.8%)  GMFCS 3 = 0  GMFCS 4 = 7 (19.4%)  GMFCS 5 = 28 (77.8%) | Not reported | Postoperative blood transfusion | Not carried out |
| McLeod et al. 2015 | Overall cohort = mean 12.7 years (SD 3.8)  Antifibrinolytic treated group = 13.1 (4.0)  Untreated group = 12.5 (3.4) | Overall cohort = 785 (51%) female  Antifibrinolytic treated group = 224 (50%)  Untreated group = 561 (51%) | Not reported | Not reported | Perioperative blood transfusion | A priori = yes  Target sample size achieved = yes |
| McNeil et al. 2020 | No antifibrinolytic group = mean 14.7 years (SD 1.6)  Antifibrinolytic group = 14.7 (1.7) | No antifibrinolytic group = 224 (74.2%) female  Antifibrinolytic group = 227 (75.2%) | Not reported | Not reported | Intraoperative blood transfusion | A priori = no  Target sample size achieved = N/A |
| Abbot et al. 2014 | Total study population = mean 13.24 years (SD 4.03) | Total study population = 79 (67.5%) female | Not reported | Not reported | Intraoperative blood loss | Not carried out |
| Bichmann et al. 2023 | Not reported | Not reported | Not reported | Not reported | Perioperative blood loss and perioperative transfusion requirement (neither specifically designated as primary outcome) | Not carried out |
| Bird et al. 2011 | Not reported | Not reported | Not reported | Loading doses varied between 2 and 100mg/kg, and intraoperative infusions varied between 3 to 10mg/kg/hr | Not stated | Not carried out |
| Chiem et al. 2017 | 15 years | Male | Not reported | Loading dose of 5mg/kg followed by 5mg/kg/hr infusion | Not stated | Not carried out |
| **Colomina et al. 2009 | Aprotinin group = mean 41.4 years (SD 17.0)  TXA group = 35.1 (SD 18.2) | Aprotinin group = 22 (73.3%) female  TXA group = 28 (71.8%)  NB: not reported separately for paediatric patients | Not reported | Bolus of 10mg/kg during 20 minutes before surgery, followed by 2mg/kg/hr infusion | Perioperative blood loss | Not carried out |
| Degiorgio-Miller et al. 2013 | Mean 13.5 years | Not reported | Spastic quadriplegia (not further specified) | Not reported | Number of revision procedures | Not carried out |
| Dhawale et al. 2012 | Antifibrinolytic group = mean 14.1 years (SD 2.4)  Non-antifibrinolytic group = 14.7 (2.8) | Antifibrinolytic group = 17 (38.6%) female  Non-antifibrinolytic group = 16 (40.0%) | Not reported | Loading dose of 30mg/kg followed by 1mg/kg/hr infusion | Intraoperative blood loss | A priori = no  Target sample size achieved = N/A |
| Dick et al. 2019 | Overall cohort = median 14.0 years [IQR 13-16] | Overall cohort = 705 (67.9%) female | Not reported | Loading dose of 30mg/kg followed by 1mg/kg/hr infusion until skin closure | Perioperative blood transfusion | Not carried out |
| Dong et al. 2021 | Not reported | Not reported | Not reported | Loading dose of 1g followed by 10mg/kg/hr infusion | Perioperative blood transfusion | Not carried out |
| Dupuis et al. 2015 | Overall cohort = mean 15 years (SD 3) | Not reported | Not reported | Bolus of 50mg/kg followed by 10mg/kg/hr infusion (maximal dose = 4g/d) | Perioperative blood transfusion | Not carried out |
| Duvernay et al. 2020 | Not reported | Not reported | Not reported | Bolus followed by infusion - doses not specified | Platelet dysfunction | Not carried out |
| Eisler et al. 2020 | Antifibrinolytic group: <8 years = 179 (3.3%), 8-12 years = 1526 (28.0%), 13+ years = 3729 (68.8%)  Non-antifibrinolytic group: <8 years = 76 (6.4), 8-12 years = 341 (28.8%), 13+ years = 775 (65.0%) | Antifibrinolytic group = 3716 (68.4%) female  Non-antifibrinolytic group = 835 (70.1%)  NB: Reported for overall group (i.e. not reported separately for neuromuscular scoliosis patients) | Not reported | Not reported | Perioperative blood transfusion | A priori = no  Target sample size achieved = N/A |
| Ezhevskaya et al. 2015 | Range 15 to 18 years | Not reported | Not reported | Bolus of 15mg/kg followed 2mg/kg/hr infusion | Intraoperative blood loss | Not carried out |
| Fernandez et al. 2021 | Mean 14.9 years (SD 9.3) | Not reported | Not reported | Not reported | Unclear (none of the outcomes designated to be the primary outcome) | Not carried out |
| Gibson et al. 2022 | Neuromuscular group = mean 12 years (range 10-17) | Neuromuscular group = 6 (66.7%) female | Not reported | Bolus: patients <20kg body mass = 100mg/kg; patients ≥20kg body mass = bolus of 2000mg  Infusion: patients <50kg body mass = 10mg/kg/hr; patients ≥50kg body mass = 500mg/hr | Intraoperative blood transfusion  Perioperative blood loss | Not carried out |
| Gurajala et al. 2013 | Mean 14.3 years (SD 3.8) | 59 (58.8%) female | Not reported | Three boluses = 10mg/kg before skin incision, 5mg/kg during instrumentation, and 5mg/kg after instrumentation | The need for early postoperative mechanical ventilation | Not carried out |
| Halanski et al. 2014 | Amicar group = mean 13.9 years (range 13.1-14.6)  TXA group = 13.2 (12.4-14.1) | Amicar group = 19 (76.0%)  female  TXA group = 17 (77.0%) | Not reported | Bolus of 100mg/kg over 30 minutes, followed by 10mg/kg/hr infusion | Intraoperative blood loss | A priori = yes  Target sample size achieved = no |
| Ivasyk et al. 2022 | Overall cohort = mean 14.1 years (SD 3.0) | Overall cohort = 1697  (64.5%) female | Not reported | Not reported | DVT, PE, seizure, or stroke | Not carried out |
| Jurgens et al. 2017 | Mean 21.7 years (range 3-71) | 38 (74.5%) female | Not reported | Not reported | Unclear (none of the outcomes designated to be the primary outcome) | Not carried out |
| Koraki et al. 2020 | Protocol group = mean 16.26 years (SD 1.23)  Non-protocol group = 15.89 (1.29) | Protocol group = 9 (52.9%) female  Non-protocol group = 9 (50.0%) | Not reported | Bolus of 30mg/kg followed by 1mg/kg/hr | Intraoperative blood transfusion | Not carried out |
| Mihas et al. 2021 | Idiopathic group (15 patients) = mean 14.7 years (SD 2.6)  Non-idiopathic group = 12.4 (0.9) | Idiopathic group = 10 (66.7%) female  Non-idiopathic group = 3 (60%) | Not reported | Bolus of 50mg/kg, followed by 5mg/kg/hr infusion.  Topical TXA also used in the form of 1:1 TXA:normal saline-soaked sponges in the wound. | Intraoperative blood loss | Not carried out |
| Neilipovitz et al. 2001 | Control group = mean 13.7 years (SD 2.5)  TXA group = 14.1 (2.1) | Control group = 13 (72.2%) female  TXA group = 10 (45.5%) | Not reported | Loading dose of 10mg/kg over 15 minutes, followed by 1mg/kg/hr infusion from end of loading dose until skin closure | Perioperative blood transfusion | A priori = no  Target sample size achieved = N/A |
| Nellis et al. 2021 | Median 14 years (IQR 12-16) | Not reported | Not reported | Not reported | Perioperative blood transfusion | Not carried out |
| O'Donoghue et al. 2020 | Not reported | Not reported | Not reported | Not reported | Intraoperative blood loss | Not carried out |
| Schur et al. 2017 | Neuromuscular scoliosis group = median 14.6 years (IQR 8-22.7) | Neuromuscular scoliosis group = 113 (46.5%) female | Not reported | Loading dose of 50mg/kg, followed by 5 to 10mg/kg/hr infusion at the discretion of the attending anaesthetist.  Table 3 of the article indicates that patients in the TXA group had a greater degree of deformity, and longer duration of surgery. | Perioperative blood transfusion | Not carried out |
| Schur et al. 2018 | Hypothermic group = median 14.4 years (IQR 13.0-16.2)  Normothermic group = 14.4 (12.8-16.5) | Hypothermic group = 143 (62.2%) female  Normothermic group = 177 (63.2%) | Not reported | Loading dose of 50 mg/kg, followed by 5 to 10mg/kg/hr infusion | Perioperative blood transfusion | Not carried out |
| Shrader et al. 2018 | One-surgeon group = mean 12.6 years (range 8-18)  Two-surgeon group = 15.5 (11-22) | Not reported | One-surgeon group: GMFCS IV = 7 (28.0%), GMFCS V = 18 (72.0%)  Two-surgeon group: GMFCS IV = 7 (28.0%), GMFCS V = 18 (72.0%) | Not reported | Total operative time and estimated intraoperative blood loss | A priori = yes  Target sample size achieved = yes |
| Singh et al. 2013 | 11 years | Female | Not reported | Loading dose of 10mg/kg, followed by 1mg/kg/hr infusion | Not stated | Not carried out |
| Soini et al. 2023 | Age at final 2-year follow-up given: neuromuscular scoliosis group = mean 18.1 years (SD 3.9)  Adolescent idiopathic scoliosis group = 17.6 (2.6) | Neuromuscular scoliosis group = 33 (55.0%) female  Adolescent idiopathic scoliosis group = 66 (55.0%) | Neuromuscular scoliosis group preoperative = mean 3.6 (SD 0.8)  Adolescent idiopathic scoliosis group = 4.0 (0.5)  NB: 'Function' reported as part of SRS-24 (higher score = better function) | Bolus of 30mg/k (max 1500mg), followed by 10mg/kg/hr (max 500mg/hr) infusion | Postoperative mean differences in health-related quality of life domains between the groups from preoperative to the 2-year follow-up | Not carried out |
| Spiessberger et al. 2023 | Overall cohort = mean 9.9 years (SD 4.4, range 1.5-18.6) | Overall cohort = 36 (46.0%) female | Not reported | Not reported | Unclear (none of the outcomes designated to be the primary outcome) | Not carried out |
| Tan et al. 2018 | Not reported | Not reported | Not reported | Not reported | Perioperative transfusion rate and transfusion volume | Not carried out |
| Vasan et al. 2021 | Fresh whole blood group = mean 14.0 years (SD 3.1)  Component group =14.9 (2.7) | Fresh whole blood group = 23 (76.7%) female  Component group = 29 (82.9%) | Not reported | 1g at induction, followed by 1g at 3-hourly intervals, up to 3g total dose. | Unclear (none of the outcomes designated to be the primary outcome) | Not carried out |
| Verma et al. 2010 | Neuromuscular scoliosis patients between 10 and 80 years of age will be included | No selective recruitment for sex reported | Not reported | Loading dose of 10mg/kg, followed by 1mg/kg/hr infusion. | Perioperative blood loss | A priori = yes  Target sample size achieved = N/A (protocol) |
| Vrbica et al. 2023 | <18 years old at time of operation | No selective recruitment for sex reported | Not reported | Loading dose of 10 to 15mg/kg. | All of the following are stated to be "primary outcomes": The incidence of any adverse events, adverse drug reactions, serious adverse events, serious adverse reactions, unexpected adverse reactions and suspected unexpected serious adverse reactions | A priori = yes  Target sample size achieved = N/A (protocol) |
| Weissman et al. 2020 | No TXA = mean 14.7 years (SD 2.2)  IV TXA = 14.6 (2.1)  Topical TXA = 14.7 (3.7)  IV and topical TXA = 15.6 (2.9) | No TXA = 29 (82.9%) female  IV TXA = 29 (89.2%)  Topical TXA = 16 (69.6%)  IV and topical TXA = 65 (75.6%) | Not reported | IV: bolus of 20mg/kg, followed by 1mg/kg/hr infusion until skin closure.  Topical: gauze for packing was soaked in solution of 6g TXA diluted in 3L saline.  “IV TXA had significantly more levels fused (12.67±3.84) than the no TXA and the topical + IV TXA groups (resp 10.56 ± 2.25 levels and 10.22±3.12 levels, p<0.001).” | The primary outcomes were perioperative blood loss, surgical time, postoperative haematocrit/haemoglobin, perioperative transfusion rates, and duration of drain insertion | Not carried out |
| Fernandes 2020 | Before multimodal blood loss reduction program = mean 13 years (SD 5)  After introduction of multimodal program = 13 (4) | Before multimodal blood loss reduction program = 52 (50.0%) female  After introduction of multimodal program = 48 (58.0%) | Not reported | Bolus of 10 to 30mg/kg, followed by 3 to 5mg/kg/hr infusion | Intraoperative estimated blood loss, haemoglobin and hematocrit levels at 4 different times, and perioperative transfusion units | Not carried out |
| **Hip studies (AND HIP COMPONENT OF LU 2020)** | | | | | | |
| Adler et al. 2022 | No intraoperative transfusion group = mean 9.8 years (SD 5.8)  Intraoperative transfusion group = 9.3 (5.8) | No intraoperative transfusion group = 60.1% female  Intraoperative transfusion group = 53.5% female | Not reported | Since January 2018 = 10mg/kg bolus followed by 5mg/kg/hr intraoperative infusion.  Prior to 2018, dosing was at the discretion of the anaesthetist. | Intraoperative transfusion | Not carried out (convenience sample (the sample size was determined by the number of patients undergoing reconstructive surgery including osteotomy for hip dysplasia at the study institution from January 2012 through April 2021))s |
| Brouwer et al. 2021 | Overall = mean 8.0 years (SD 4.3)  No TXA group = 8.0 (4.3)  Pre-op TXA group = 6.7 (4.9)  Intra-op TXA group = 8.4 (3.8)  TXA pump group = 6.7 (4.2)  Pre- and intra-op TXA (single patient) = 11.1 | Overall = 234 (68.8%) female  No TXA group = 186 (69.1%) female  Preoperative TXA group = 12 (60.0%) female)  Intraoperative TXA group = 28 (65.1%) female  TXA pump group = 7 (100%) female  Preoperative + intraoperative TXA patient = female | Not reported | Preoperative TXA group mean dosage = 16mg/kg (SD 7)  Intraoperative TXA group mean dosage = 19mg/kg (SD 7)  Intraoperative TXA pump group mean dosage = 50mg/kg (SD 43)  Preoperative + intraoperative TXA patient = 27mg/kg  “patients who received TXA during surgery (intraoperatively), most likely received this as a response on intraoperative course and observed (substantial) blood loss. This might lead to confounding by indication when assessing the association between TXA and blood loss” | Intraoperative blood loss | Not carried out |
| Lu et al. 2020 | SEMLS group = mean 8.8 years (SD 3.3) | SEMLS group = 38 (38.8%) female | GMFCS 1 = 2 (2.0%)  GMFCS 2 = 23 (23.5%)  GMFCS 3 = 22 (22.4%)  GMFCS 4 = 21 (21.4%)  GMFCS 5 = 30 (30.6%) | Not reported | Postoperative blood transfusion | Not carried out |
| Compton et al. 2022 | Overall cohort = mean 9.4 years (SD 3.8) | Non-TXA group = 110 (35%) female  TXA group = 42 (52%) female | Non-TXA group: GMFCS 1 = 6 (1.9%), GMFCS 2 = 33 (10.6%), GMFCS 3 = 40 (12.9%), GMFCS 4 = 125 (40.3%), GMFCS 5 = 106 (34.2%)  TXA group: GMFCS 1 = 1 (1.3%), GMFCS 2 = 9 (11.3%), GMFCS 3 = 10 (12.5%) , GMFCS 4 = 33 (41.3%), GMFCS 5 = 27 (33.8%) | Earlier in the study period, patients typically received a loading dose of 50mg/kg, followed by a maintenance dose of 5 to 10mg/kg/hr.  Later in the study period, patients typically received loading dose is 15 to 20mg/kg, followed by maintenance of 5mg/kg/hour for the remainder of surgery.  ” Patients in the TXA group (62.5%; 50/80) were more likely to use preoperative seizure medications than patients in the No-TXA group (41.0%; 127/310) (P=.001; Table 1). Patients in the TXA group (83.8%; 67/80) were more likely to undergo a bilateral VDRO than patients in the No-TXA group (68.7%; 213/310) (P=.008; Table 1).” | Perioperative transfusion rate | Not carried out |
| Tzatzairis et al. 2020 | Unilateral VDRO TXA group = mean 6.5 years (SD 4.8)  Unilateral VDRO non-TXA group = 8.0 (5.3)  Bilateral VDRO TXA group = 6.6 (4.9)  Bilateral VDRO non-TXA group = 7.8 (5.3) | Unilateral VDRO TXA group = 9 (56%) female  Unilateral VDRO non-TXA group = 7 (54%) female  Bilateral VDRO TXA group = 9 (69%)  Bilateral VDRO non-TXA group = 5 (56%) female | Unilateral VDRO TXA group: GMFCS II = 5 (31.3%), GMFCS III = 9 (56.3%), GMFCS IV = 2 (12.5%), GMFCS V = 0  Unilateral non-TXA group: GMFCS II = 2 (15.4%), GMFCS III = 6 (46.2%), GMFCS IV = 5 (38.5%), GMFCS V = 0  Bilateral TXA group: GMFCS II = 0, GMFCS III = 5 (38.5%), GMFCS IV = 7 (53.8%), GMFCS V = 1 (7.7%)  Bilateral non-TXA group: GMFCS II = 1 (11.1%), GMFCS III = 3 (33.3%), GMFCS IV = 4 (44.4%), GMFCS V = 1 (11.1%) | Bolus of 15 mg/kg (max 1g) followed by 10 mg/kg/hr infusion until the end of the operation. | Perioperative transfusion rate and perioperative total blood loss (both stated to be primary aims) | Not carried out |
| Nazareth et al. 2018 | Not reported (although stated to be similar between TXA and non-TXA groups) | Non-TXA group = 89 (40.1%) female  TXA group = 15 (41.7%) | Non-TXA group: GMFCS II = 26 (11.7%), GMFCS III = 34 (15.3%), GMFCS IV = 106 (47.7%), GMFCS V = 56 (25.2%)  TXA group: GMFCS II = 2 (5.6%), GMFCS III = 4 (11.1%), GMFCS IV = 19 (52.8%) , GMFCS V = 11 (30.6%) | Loading dose of 50 mg/kg followed by 5 mg/kg/hour to 10 mg/kg/hour infusion at the discretion of the anaesthesiologist. | Transfusion rates, complication rates, and haemoglobin levels (all three stated as primary aims) | A priori = no  Target sample size achieved = N/A |
| Dowlut et al. 2017 | Not reported | Overall cohort = 19 (57.6%) female | Not reported | Not reported | Unclear (none of the outcomes designated to be the primary outcome) | Not carried out |
| Lins et al. 2020 | TXA group = mean 10 years (SD 4.4)  Non-TXA group = 9.1 (3.8) | TXA group = 17 (36.2%) female  Non-TXA group = 30 (41.7%) | TXA group: GMFCS I = 1 (2%), GMFCS II = 2 (4%), GMFCS III = 7 (15%), GMFCS IV = 15 (32%), GMFCS V = 22 (47%)  Non-TXA group: GMFCS I = 0, GMFCS II = 14 (19%), GMFCS III = 10 (14%), GMFCS IV = 22 (31%), GMFCS V = 26 (36%) | Bolus of 10 to 30mg/kg over 15 minutes, followed by 5 to 10mg/kg/hr infusion  “It was found that subjects with a higher GMFCS level were more likely to receive TXA such that for each additional GMFCS level, the odds of receiving TXA increased by 44% (OR, 1.44; 95% CI, 1.10-1.91; P=0.009). Similarly, the number of comorbid factors increased the likelihood of TXA such that for each additional comorbid factor the odds of TXA increased by 54% (OR, 1.54; 95% CI, 1.11-2.14; P=0.009). It was also determined that the subjects undergoing hip surgery which involved the pelvis had 2.6 times the odds for receiving TXA (OR, 2.59; 95% CI, 1.27-5.28; P=0.009) compared with those undergoing femoral treatment alone” | Unclear (none of the outcomes designated to be the primary outcome) | A priori = no  Target sample size achieved = N/A |
| Majid et al. 2015 | Non-TXA group = mean 10.2 years (SD 3.3)  TXA group = 9.8 (3.3) | Non-TXA group = 16 (43.2%) female  TXA group = 9 (64.3%) | Non-TXA group = 27 (75.0%) GMFCS ‘high’  TXA group = 11 (78.6%)  NB: 'GMFCS (low/high)' not defined in the manuscript | Not reported | Perioperative transfusion rate | A priori = no  Target sample size achieved = N/A (but no, according to post hoc calculation) |
| Masrouha et al. 2022 | TXA group = mean 9.1 years (range 4.3-12.8)  Non-TXA group = 11.1 (5.0-17.1) | TXA group = 13 (54.2%) female  Non-TXA group = 11 (57.9%) | TXA group: GMFCS I-III = 7 (29%), GMFCS IV-V = 17 (71%)  Non-TXA group: GMFCS I-III = 4 (21%), GMFCS IV-V = 15 (79%) | Loading dose of 10 or 50mg/kg followed by 1 or 5mg/kg/hr, at the discretion of the anaesthetist. | Unclear (none of the outcomes designated to be the primary outcome) | Not carried out |
| van Kouswijk et al. 2023 | Range 1-18 years | No selective recruitment for sex reported | Not reported | Bolus of 15 mg/kg | Intraoperative blood loss | A priori = yes  Target sample size achieved = N/A (protocol) |
| Zuccon 2023 | TXA group = mean 10.3 years (SD 2.6)   Control group = 10.7 (SD 3.9) | Only reported for overall cohort = 11 (35.5%) | Overall cohort: GMFCS I = 1 (3.2%), GMFCS IV = 10 (32.2%), GMFCS V = 20 (64.5%) | Bolus of 10mg/kg | Haemoglobin reduction rate (24 h preoperative minus 24 h postoperative), days in intensive care unit, total length of hospital stay, volume of intraoperative blood loss, and in-hospital complications | A priori = no  Target sample size achieved = N/A (but no, according to post hoc calculation |
| *presented as mean SD in years, and presented separately for neuromuscular/cerebral palsy patients, wherever possible - limited by what was reported in the included records, including which groups had summary statistic reported for age; **Age range of overall cohort = 10 to 75 years, with neuromuscular scoliosis being in a separate category to ‘adult scoliosis’ in the manuscript; CP = cerebral palsy; SD = standard deviation; IQR = interquartile range; TXA = tranexamic acid; SEMLS = single event multilevel surgery; VDRO = varus derotational osteotomy; GMFCS = Gross Motor Function Classification System; SRS-24 = Scoliosis Research Society 24-item questionnaire; DVT = deep vein thrombosis; PE = pulmonary embolism | | | | | | |

## Table S5: Patient and intervention characteristics – summarised

| **Characteristic** | **Findings - spine (n = 41 studies)** | **Findings - hip (n = 12 studies)** |
| --- | --- | --- |
| Sample size calculation | A priori = ***6 (14.6%)   - Target sample size achieved (for studies with a priori sample size calculation) = 3 (75.0%) of 4   Post hoc = 4 (9.8%)  Not carried out = 31 (75.6%) | A priori = 1 (8.3%)   - Target sample size achieved (for studies with a priori sample size calculation) = N/A (RCT protocol) (8.3%)   Post hoc = 4 (33.3%)  Not carried out = 7 (58.3%) |
| **Primary outcome | Blood loss:   - Intraoperative = 9 (20.5%) - Postoperative = 0 - Perioperative = 4 (9.1%)   Transfusion:   - Intraoperative = 3 (6.8%) - Postoperative = 1 (2.3%) - Perioperative = 13 (29.5%)   Other = 6 (13.6%)  Unclear or not stated = 8 (18.2%) | Blood loss:   - Intraoperative = 3 (20.0%) - Postoperative = 0 - Perioperative = 1 (6.7%)   Transfusion:   - Intraoperative = 1 (6.7%) - Postoperative = 1 (6.7%) - Perioperative = 4 (26.7%)   Other = 2 (13.3%)  Unclear = 3 (20.0%) |
| TXA given intravenously unless otherwise specified; *one study included a bolus-only arm and a bolus + infusion arm; **some studies stated more than one primary outcome (denominator for spine studies = 44; denominator for hip studies = 15); ***includes 2 randomised controlled trial protocols and final sample size not yet known; GMFCS = Gross Motor Function Classification System; SRS-24 = Scoliosis Research Society 24-item questionnaire | | |

## Table S6: Findings of studies with a primary aim related to TXA

| **Record ID** | **Blood loss measurement technique** | **Funding source (external/non-institutional)** | **Main findings** | **Secondary findings** |
| --- | --- | --- | --- | --- |
| **Spine studies** | | | | |
| Sethna et al. 2005 | Estimated blood loss determined hourly from surgical suction and autotransfusion system reservoirs, and by weighing sponges from the operative field. | Institutional/departmental sources | Blood loss: Multiple stepwise linear regression analysis indicated that three variables were independently predictive of blood loss: preoperative platelet count, ASA physical status, and treatment with TXA. Treatment with TXA significantly reduced intraoperative blood loss (P < 0.001).  Transfusion: Mean total transfusion volume in secondary scoliosis patients was significantly lower in the TXA patients compared with the placebo patients: mean 808mL (SD 531) vs 1391 (723), p = 0.04. | Haematoma: not reported  Length of stay: not reported  Infection: not reported  Other: duration of surgery did not differ between groups (TXA vs no TXA) |
| McNeil et al. 2020 | Not applicable (blood loss was not measured in this study) | None for this study | Blood loss: N/A  Transfusion: There was no significant difference in intraoperative transfusion between patients who received antifibrinolytic drugs and those who did not: OR = 0.71 (99% CI = 0.40 to 1.26, P = 0.12). There was no difference in median cell saver volume 114mL (0, 250 mL) compared to 100mL (0, 246 mL), P = 0.04. There were no differences in postoperative allogeneic transfusion: OR = 1.23, (99% CI = 0.54 to2.81, P = 0.52) or the percentage of patients requiring massive transfusion OR = 1.0, (99% CI = 0.34 to 2.92, P = 1.0). There were no apparent reductions in intraoperative transfusion in any subgroup of patients who received antifibrinolytic drugs. | Haematoma: not reported  Length of stay: not reported  Infection: not reported  Other: operative time no antifibrinolytic group = mean 254.1 minutes (SD 85.3), antifibrinolytic group = 265.4 (76.6), p = 0.14 |
| Bichmann et al. 2023 | Not reported | Not reported | Blood loss: Estimated blood loss (p = 0.531) did not differ significantly. TXA administration had a significant negative effect on estimated blood loss (B = -510.98, p = 0.020).  Transfusion: Administration of allogeneic packed erythrocytes (p = 0.508) did not differ significantly. | Haematoma: not reported  Length of stay: not reported  Infection: postoperative complications including systemic inflammatory response syndrome (p = 0.818), pneumonia (p = 0.628), and urinary tract infection (p = 0.628), did not differ significantly between the two groups  Other: postoperative Hb-levels were significantly higher in the TXA-group (9.13 ± 1.27 g/dL) compared with the No-TXA-group (8.41 ± 1.47 g/dL) on the first postoperative day (p = 0.038). Surgical duration (p =  0.009) was significantly higher in the TXA-group. |
| **Colomina et al. 2009 | Intraoperative blood loss was measured by aspiration and by weighing the used sponges and gauze on a digital scale.  Postoperative blood loss was measured as the volume through the drains during the first 48 postoperative hours.  Total blood loss was calculated as the sum of intraoperative and postoperative blood loss. | Not reported | Blood loss:  There were no significant differences in the intraoperative (mean 2118.3mL (SD 1255.8) vs 1608.7 (1012.8), p = 0.066), postoperative (1227.2 (623.5) vs 1017.2 (776.8), p = 0.230) or total blood loss. Intraoperative blood loss in the aprotinin group was almost 700 mL greater than in the tranexamic acid group: mean 3312.2mL (SD 1523.9) vs 2627.2 (1386.3), (p = 0.056). Intraoperative blood loss relative to blood volume of each patient was 0.42 (42%) in the tranexamic acid group and 0.55 (55%) in the aprotinin group. A larger (although nonsignificant) difference was observed when total blood loss was calculated according to blood volume: tranexamic acid group 0.69 and aprotinin group 1.46 (p-value not reported). Intraoperative and total blood loss per fusion level was significantly lower in the aprotinin group than in patients receiving tranexamic acid (228.5 (187.5) vs 428.1 (494.7), p = 0.025). There were no differences between the groups in the intraoperative blood loss per hour of surgery ((mL per hour surgery) = 192.5 (81.6) vs 216.7 (118.9), p = 0.3).  The multivariable analysis identified sex (p = 0.005), age (p = 0.001) and surgery duration (p = 0.001) as independent variables related with blood loss in spine surgery. Type of antifibrinolytic was not statistically significant (p = 0.07).  Transfusion:  Aprotinin patients compared to TXA patients received more autologous blood units (2.2 (2.1) vs 1.3 (1.5), p = 0.047)  and more total units (4.1 (2.1) vs 2.6 (2.2), p = 0.008).  There were no differences between Aprotinin and TXA groups in terms of allogeneic units transfused (1.8 (2.6) vs 1.3 (2.3), p = 0.37), transfused units per hour of surgery (mL per hour surgery = 0.34 (0.75) vs 0.38 (0.36), p = 0.4), nor transfused units per level fused (mL per level = 0.43 (0.26) vs 0.53 (0.75), p = 0.4).  In the multivariable model, the variables significantly associated with the number of transfused units were age (p = 0.013) and duration of surgery (p = 0.001). Again, the type of antifibrinolytic given was not significant (p = 0.4) | Haematoma: Not reported  Length of stay: Aprotinin group vs TXA group = 17.2 days (SD 10.3) vs 13.2 (8.8), respectively (p = 0.086).  Infection: Not reported  Other: Postoperative haemoglobin and haematocrit were similar in Aprotinin vs TXA group: mean  postoperative haemoglobin = 9.9g/dL (SD 1.1) vs 10.3 (1.1), p = 0 .236; postoperative haematocrit = mean 29.9% (SD 3.4) vs 29.8 (3.0), p = 0.900.  Duration of surgery between Aprotinin and TXA groups = 662.3 mins (SD 210.6) vs 448.4 (157.2), respectively (p = 0.001. None of the patients in either group experienced complications related with the administration of antifibrinolytic drugs including thromboembolic events, renal insufficiency, cardiac complications or anaphylactic phenomena |
| Ezhevskaya et al. 2015 | Not reported | Not reported | Blood loss: The main effect of reducing intraoperative blood loss by 65% (524 ml, p=0.001) was obtained in group 1 [TXA and epidural anaesthesia] as compared with group 3 [general anaesthesia, no TXA].  In patients without epidural anaesthesia, TXA decreased blood loss to 29.7% (p=0.01) compared with group 4. The use of epidural anaesthesia without TXA reduced intraoperative blood loss to 50% (730 ml, p=0.005)  Transfusion: Not reported | Haematoma: Not reported  Length of stay: Not reported  Infection: Not reported  Other: There were no complications in any group. |
| Gibson et al. 2022 | Blood loss was calculated in two ways.  First, using a hematocrit-based formula for estimated red cell mass deficit normalised as a percent of total blood volume and normalised blood product transfused, calculated as volume of intraoperative blood product (allogeneic and autologous), normalised to patient weight.  Second, the anaesthesia postoperative record of estimated blood loss was also noted from patient charts (NB: no details were provided on how this was estimated). | Study was supported by Caitlin Lovejoy Fund, the Vanderbilt University Medical Center Department of Orthopaedics (JGS), the Katherine Dodd Faculty Scholar Fund (AJB), the  Vanderbilt Center for Musculoskeletal Research Faculty Award (MTD), the Vanderbilt University School of Medicine Research Immersion Program (LJM), UL1 TR002245 (MTD) and NHLBI-F31HL149340-02 (BHYG). | Blood loss: The mean estimated red cell mass deficit was 17.9% total blood volume (range: 7.7–51.8). On average, patients with idiopathic scoliosis lost less blood than those with neuromuscular scoliosis (17.0 vs 19.7% TBV, respectively).  The magnitude of the intra-operative increase in plasmin activation correlated with normalised blood product transfusion (R2 = 0.388, p = 0.007) and ERCM defcit (R2 = 0.400, p = 0.006). Furthermore, the intra-operative increase in D-dimer weakly correlated with estimated red cell mass deficit (R2 = 0.264, p = 0.035).  BMI and number of osteotomies were not associated with estimated red cell mass deficit (R2 = 0.109, 0.005, p = 0.201, 0.775, respectively), and there were no significant differences in total blood loss measurements between the two surgeons performing the procedures (p = 0.475).  The number of levels fused during the procedure was weakly associated with estimated red cell mass deficit (R2 = 0.308, p = 0.02).  While the TXA dosing varied based on patient weight, there was no association between TXA dosing per kilogram of body weight and estimated red cell mass deficit for either bolus (R2 = 0.190, p = 0.09) or continuous (R2 = 0.162, p = 0.100) administration.  Transfusion: The mean normalised blood product transfused was 6.0 mL/kg (range: 0–17.9).  Across all 17 patients, 16/17 (94.1%) received autologous red blood cell salvage during the procedure, and 3/17 (17.6%) received allogeneic packed red blood cell transfusion. | Haematoma: Not reported  Length of stay: Overall = mean 4.7 days (SD 1.9)  Infection: Not reported  Other: N/A |
| Halanski et al. 2014 | Blood loss was monitored in three ways.  First, standard estimated blood loss was recorded from the medical record by estimates made in the operating room by anaesthetist and/or surgeon inspecting wound, drapes, drain canisters, sponges, etc.  Second, blood loss into the Cell Saver system was collected in addition to weighing sponges. Calculated blood loss was then determined by subtracting thee volume of heparinised saline from the total volume in the Cell Saver system, and blood-soaked sponges from averaged dry sponge weight - these two values were then summed.  Third, preoperative and postoperative haemoglobin and intraoperative blood products given to the patient were recorded. | None for this study | Blood loss:  Neuromuscular scoliosis patients (Amicar vs TXA):  Estimated blood loss (mL) = 1780 (95% confidence interval 246 to 3314) vs 630 (274 to 986), p 0.106  Calculated blood loss (mL) = 2740 (148 to 5331) vs 823 (248 to 1397), p 0.109  Blood loss/kg (mL/kg) = 70.2 (4.5 to 135.8) vs 24.2 (9.3 to 39.2), p 0.125  Blood loss/levels fused (mL/level) = 177 (7 to 346) vs 55 (16 to 95), p 0.117  Transfusion:  Transfusion rate (%) = 40% vs 20%, p 1.000 | Haematoma: Not reported  Length of stay: Not reported  Infection: Not reported  Other: N/A |
| Ivasyk et al. 2022 | Not applicable (blood loss was not measured in this study) | Ms. Ivasyk was supported by a Medical Scientist Training Program grant from the National Institute of General Medical Sciences of the National Institutes of  Health under award number T32GM007739 to the Weill Cornell/Rockefeller/  Sloan-Kettering Tri-Institutional MD- PhD Program | Blood loss: Not applicable (blood loss was not measured in this study)  Transfusion: Not reported | Haematoma: Not reported  Length of stay: Not reported  Infection: Not reported  Other: There were no reported new onset seizures or PE in patients who received TXA perioperatively, and no statistically significant difference in incidence of DVT.  No strokes were reported in either cohort. There were also no cases of in-hospital mortality.  Overall, new diagnosis of DVT, PE, stroke, and seizure were rare, with no statistically significant difference between the two groups (p=0.71, Table 2). The rate of any complication for patients not receiving TXA was 0.54% (95% CI, 0.31% to 0.94%). The odds ratio of complications in the group that received TXA compared to the group that did not was 0.46 (95% CI, 0.04 to 2.9). |
| Neilipovitz et al. 2001 | Estimated red cell mass deficit, and haemoglobin mass loss (formulae not further specified) | This study was funded by an unrestricted grant from Pharmacia  Pharmaceuticals | Blood loss: The intraoperative blood loss in the TXA group was not significantly different than in the Control group: mean 2453mL (SD 1526) vs 2703 (1292), p = 0.58.  Transfusion:  Total amount of blood transfused in TXA group vs control group = mean 1254mL (SD 884) vs 1784 (773), p = 0.045.  Analysis of covariance model (weight, scoliosis form, and treatment group) found that TXA had a significant impact on total blood transfused (p = 0.028). | Haematoma: Not reported  Length of stay: Not reported  Infection: Not reported  Other: Six patients in each group received at least 1 U of blood at a hemoglobin value above threshold. The most common reason was acute blood loss necessitating treatment before the results of the hemoglobin levels became available. Other reasons included persistent tachycardia (2 U for a TXA patient) and the desire to use up autologous blood (one in each group). A post hoc analysis was performed after the volume of these 14 blood units was subtracted from the total blood transfused values. The TXA group received significantly less total blood compared with the Control group (P 5 0.04).  The haemoglobin levels measured at the end of surgery and on the first postoperative day were similar.  TXA was well tolerated by all subjects in the treatment group. There were no cases of hemodynamic instability, clinically overt thrombotic complications, or other adverse effects associated with its use. The duration of mechanical ventilation in the intensive care unit for the TXA group and the control group was similar: mean 5.4 hours (SD 9.2) vs 7.3 (12.0), p = 0.57.  The total time spent in the intensive care unit for the TXA group and the control group was similar: mean 28.8 hours (SD 13.7) vs 32.2  (22.1), p = 0.58). |
| Schur et al. 2017 | Blood loss was estimated based on experienced surgeon and anaesthetist observation, the amount of blood in the suction canister, the weight of the lap sponges, the amount in the Cell Saver, and the first postoperative haematocrit. | Mathew Schur gratefully  acknowledges financial support from the Wright  Foundation. Gideon Blumstein gratefully acknowledges  financial support from the Meira and Shaul G. Massry  Foundation | Blood loss: Mean estimated blood loss for neuromuscular scoliosis patients in the different treatment groups was: no TXA or intrathecal morphine = mean 22.6 ml/kg (SD 3.3), intrathecal morphine only = 26.8 (2.5), TXA only = 24.9 (3.2), XA and intrathecal morphine = 24.7 (2.0). P-values were not reported but authors stated that these differences were not statistically significant.  Transfusion: 72% (174/243) of the neuromuscular scoliosis patients received packed red blood cell transfusion intraoperatively or postoperatively. In a multivariable analysis, neuromuscular scoliosis patients who received a combination of TXA and intrathecal morphine had a 79% lower odds of receiving transfusion (odds ratio 0.2, 95% confidence interval 0.06 to 0.77, p = 0.02). Neuromuscular patients who received TXA without intrathecal morphine (0.70, 0.17 to 3.00, 0.64, or intrathecal morphine without TXA (0.47, 0.13 to 1.71, 0.25), did not demonstrate reduction in odds of transfusion. | Haematoma: Not reported  Length of stay: Not reported  Infection: Not reported  Other: No complications were observed from either TXA or intrathecal morphine |
| Verma et al. 2010 | Intraoperative blood loss was estimated in the operating room, but also calculated taking into account patient body mass pre- and postoperative haematocrit, and relative fluid balance. Three papers cited: Verma K, Vecchione D, Dean L, Auerbach J, Lonner B: Reduction of MAP during surgical exposure safely reduces operative blood loss and transfusion requirements. Proceedings of the International Meeting on Advanced Spine Techniques (IMAST) 2009.; Meunier A, Petersson A, Good L, Berlin G: Validation of a haemoglobin dilution method for estimation of blood loss. Vox Sang 2008, 95(2):120-4; Howe C, Paschall C, Panwalkar A, Beal J, Potti A: A model for clinical estimation of perioperative hemorrhage. Clin Appl Thromb Hemost 2003, 9(2):131-5.  Postoperatively, blood loss was estimated through monitoring of subfascial Hemovac drain outputs at the incision site. | Not reported | Blood loss: N/A (RCT protocol, and findings have not been reported since publication for the neuromuscular scoliosis patients)  Transfusion: N/A (not reported) | Haematoma: N/A (protocol)  Length of stay: N/A (protocol)  Infection: N/A (protocol)  Other: N/A (protocol) |
| Weissman et al. 2020 | Intraoperative bleeding was determined by measuring the amount of blood collected in the suction canister and subtracting the amount of irrigation saline used, including in the sponges for packing. These sponges were crushed to remove all liquid and weighed along with compresses used during surgery.  The drain debit was measured every 12 hours and recorded in the patient’s chart. In the first 24 postoperative hours, the drain was left with negative pressure and posteriorly at free fall.  The total blood loss was defined as the sum of the intraoperative bleeding and the total drain output. | None for this study | Blood loss: For patients with neuromuscular scoliosis, there was no significant difference by TXA groups in postoperative bleeding  Transfusion: Not reported separately for neuromuscular scoliosis patients | Haematoma: Not reported  Length of stay: Not reported  Infection: Not reported separately for neuromuscular scoliosis patients  Other: N/A |
| **Hip studies** | | | | |
| Brouwer et al. 2021 | At the end of each surgery, the anaesthesiologist and surgeon agreed upon EBL based on inspection of the gauzes and suction device. | None for this study | Blood loss: In a multivariable mixed model, a single preoperative TXA bolus was associated with less estimated blood loss compared with no TXA: regression coefficient = -66 (95% confidence interval -129 to 05, p = 0.04), interpreted as the preoperative TXA group had, on average, 66mL less blood loss than the no TXA group.  Regarding the TXA subgroups, compared with no TXA: there was decreased blood loss in the ‘TXA pump’ group (n = 7), but without statistical significance (regression coefficient = -25; 95% CI -125 to 75; p = 0.62); he ‘TXA intraop’ (n = 43) and ‘TXA pre- & intraop’ (n = 1) groups were associated with increased blood loss (RC = 38; 95% CI -9 to 84; p = 0.11).  Transfusion: Not reported | Haematoma: Not reported  Length of stay: Not reported  Infection: Not reported  Other: N/A |
| Compton et al. 2022 | Blood loss was calculated according to the haemoglobin balance method (formula provided in the manuscript, which accounted for transfused blood). Preoperative haemoglobin and haematocrit values were measured within 30 days of the procedure. At the authors’ institution, there was no standard protocol requiring postoperative laboratory monitoring - patients with low estimated blood loss relative to their total blood volume, and good clinical and haemodynamic stability, did not routinely undergo blood draws postoperatively. | None for this study | Blood loss:  There was no statistical difference in estimated blood loss (EBL) as recorded in the anaesthesia records (TXA: mean 142.9mL (SD 113.1) versus No-TXA: 177.4 (169.1); p = 0.09). Patients with both preoperative and immediate postoperative haemoglobin measurements had a significantly higher EBL as recorded in the anaesthesia records (209.3 (174.6)) than patients without postoperative haemoglobin measurements (126.9 (128.3)) (p < 0.001). There was no difference in EBL between the TXA and No-TXA groups as determined via the haemoglobin balance method (TXA: 630.1 (500.7) vs No-TXA: 792.8 (515.5); p = 0.07.  There was no difference in percentage of blood loss based on total body weight (TXA: mean 9.2% (SD 7.0) vs No-TXA: 10.1 (8.7); p = 0.40. Postoperative haemoglobin and haematocrit levels were similar between the two groups (p = 0.87 and p = 0.69, respectively). The change in preoperative to postoperative haemoglobin was not significantly different between those receiving TXA (mean -3.8 g/dL (SD 1.8)) and those not receiving TXA (3.9 (2.0)) (p = 0.85). The change in preoperative to postoperative haematocrit was not significantly different between those receiving TXA (11.4 (5.1)) and those not receiving TXA (11.8 (4.7)) (p= 0.58).  Transfusion: The overall transfusion rate over the peri- and postoperative period was significantly lower in the TXA group (13.8%; 11/80) than in the No-TXA group (25.2%; 78/310) (p = 0.04). Patients administered TXA were 0.55 times as likely to undergo transfusion over the peri- and postoperative period compared to patients who did not receive TXA (risk ratio: 0.55; 95% CI: 0.31 to 0.98; p = 0.03). Therefore, the number needed to treat (NNT) with TXA to prevent a perioperative or postoperative transfusion in this series is 9.  When controlling for unilateral vs bilateral VDROs, pelvic osteotomies, and ambulatory status, TXA administration was associated with a decreased overall transfusion rate (odds ratio (OR): 0.45; 95% CI: 0.21 to 0.96; p = 0.04).  The intraoperative transfusion rates were similar between the 2 groups (TXA: 7.5% vs No-TXA: 10.3%; p = 0.53; Table 2). Patients were not less likely to undergo an intraoperative transfusion with TXA administration (RR: 0.73; 95% CI: 0.31 to 1.7; p = 0.45).  The postoperative transfusion rate was significantly lower in the TXA group (7.5%; 6/80) than in the No-TXA group (18.4%; 57/310) (p = 0.02). Patients administered TXA were 0.41 times as likely to receive a postoperative transfusion than patients who were not administered TXA (RR: 0.41; 95% CI: 0.18 to 0.91; p = 0.02). Therefore, the NNT with TXA to prevent 1 postoperative transfusion in this series is 10. When controlling for unilateral versus bilateral VDROs, pelvic osteotomies, and ambulatory status, TXA administration was associated with a decreased postoperative transfusion rate (OR: 0.35; 95% CI: 0.14 to 0.89; p = 0.03). | Haematoma: Not reported  Length of stay: Length of inpatient hospital stay was similar between the 2 groups (TXA: mean 2.9 (SD 3.0) days vs No-TXA: 2.9 (3.2); p = 0.83).  Infection: Not reported  Other: No major adverse events were associated with TXA use throughout the follow-up period (including stroke, deep vein thrombosis, pulmonary embolism) |
| Tzatzairis et al. 2020 | Total blood loss was calculated as the sum of calculated blood loss (based on haemoglobin difference) plus the volume of transfused blood.  Formulae provided in the manuscript. | None for this study | Blood loss: Both the unilateral TXA and the bilateral TXA groups had less total blood loss. In the unilateral group, the TXA subgroup had a mean TBL of 241 ml (SD 72 ml) compared to 369 ml (SD 172) in the non-TXA group, p = 0.045. Similar results (p = 0.047) were obtained from the bilateral group [287 ml (SD 114) of TBL in the TXA subgroup vs. 467 ml (SD 210) in the non-TXA subgroup].  Transfusion: Both the unilateral TXA and the bilateral TXA groups had lower transfusion rates compared to the non-TXA groups. Statistically significant differences noted regarding the transfusion rates in the unilateral group (TXA group 25% vs non-TXA group 61.5%, p = 0.035). In the bilateral group, the rates were 69.2% and 88.8%, respectively (p = 0.069). However, the use of TXA (TXA subgroup) seems to have dramatically reduced both the transfusion percentages and the quantity of the transfused units during the bilateral VDROs. | Haematoma: Not reported  Length of stay: Unilateral VDRO TXA group vs non-TXA group = mean 4.7 days (SD 3.2) vs 5.3 (3.5) (p = 0.356). Bilateral VDRO TXA group vs non-TXA group = 6.3 (4.2) vs 6.2 (4.1) (p = 0.789)  Infection: Not reported  Other: Pre- and post-operative Hb values were similar between groups: pre-op Hb unilateral VDRO TXA vs non-TXA = mean g/dL 11.8 (0.7) vs 11.9 (0.9), p = 0.779, pre-op Hb bilateral VDRO TXA vs non-TXA = 12.0 (0.9) vs 11.8 (1.0), p = 0.66; post-op lowest Hb unilateral VDRO TXA vs non-TXA = 8.8 (0.5) vs 8.9 (0.75), p = 0.546, post-op lowest Hb bilateral VDRO TXA vs non-TXA = 9.1 (0.8) vs 8.6 (0.7), p = 0.15.  No adverse events or complications related to TXA administration were documented during the post-op period.  Operation duration: Unilateral VDRO TXA group vs non-TXA group = mean 165 mins (SD 24) vs 175 (24), p = 0.292. Bilateral VDRO TXA group vs non-TXA group = 238 (15) vs 244 (14), p = 0.4. |
| Nazareth et al. 2018 | At the conclusion of each surgery, anaesthesiologist and surgeon agreed upon an estimated blood loss by inspecting surgical sponges and suction. | None for this study | Blood loss: There was no statistical difference in estimated blood loss (TXA: mean 144.4 mL (range 25 to 400, SD 102.2) versus No TXA:159.0 (10 to 850, 144.8), p = 0.58) or percentage blood loss based on total body weight (TXA: 8.9% versus No TXA: 9.2%, p = 0.83).  Transfusion: No significant differences in total transfusion rates over the peri- and postoperative period were seen between groups (TXA: 11.1 % versus No TXA: 19.8%, p = 0.21). Intraoperative (TXA: 2.8% versus No TXA: 9.0%, p = 0.20) and postoperative blood product transfusion rates were not statistically different between groups (TXA: 8.3% versus No TXA: 14.4 %, p = 0.32). | Haematoma: Not reported  Length of stay: Length of inpatient hospital stay was not significantly different between groups (TXA: 3.0 days (1 to 17, sd 3.2), No TXA: 2.7 days 1 to 19, sd 2.4), p = 0.47).  Infection: Not reported  Other: Postoperative haemoglobin difference (TXA: -3.6 g/dL (-7.2 to -0.4, SD 1.5) versus No TXA: -3.8 g/dL -8.8 to -0.2, SD 2.2), p = 0.76) and haematocrit difference (TXA: -10.7 (-20.5 to -0.3, SD 3.9) versus No TXA: -11.4 (-25.6 to 0.8, SD 5.0), p = 0.51) from baseline were similar between groups.  No adverse events associated with TXA administration (including stroke or deep vein thrombosis) occurred within the follow-up period. |
| Dowlut et al. 2017 | Haemoglobin drop (not further specified) | Not reported | Blood loss: There was no significant difference (p>0.05) in post-operative Hb drops between the TXA group and the non-TXA group.  Transfusion: In current audit, the percentage of patients receiving cell salvage and transfusions were higher in the TXA group compared to the non-TXA group. | Haematoma: Not reported  Length of stay: Not reported  Infection: Not reported  Other: A general trend was observed that for more extensive surgery, TXA was more likely to be administered. 61% of patients received intraoperative TXA, 21% did not. Since our previous audit, the number of patients receiving TXA had increased from 39% to 74%. |
| Lins et al. 2020 | Estimated blood loss (intraoperatively) was reported by the anaesthetist and surgeon at the end of surgery.  Total blood loss was calculated based on haematocrit value at the end of the case and at the lowest recorded haematocrit value, which was either intraoperative or postoperative during their admission. The formula for total blood loss is provided in the manuscript and incorporated a measure of estimated blood volume (80cc/kg). | None for this study | Blood loss: The calculated intraoperative percent loss of estimated blood volume was statistically similar between the groups: 24.7 (12.62) TXA group vs 28.3 (12.27) non-TXA group, p = 0.20. The calculated perioperative percent loss of EBV was less for those who were given TXA compared with those who were not: 36.9 (9.60) vs 42.7 (9.95), p = 0.001.  There was no difference detected in reported EBL (median= 200 mL for both groups (interquartile range TXA group = 138 to 400, non-TXA group = 150 to 300; p = 0.63)  Transfusion:  The estimated odds of intraoperative transfusion for those administered TXA compared with those not administered TXA was 1.37 (95% CI, 0.53-3.58; p = 0.52) indicating no detectable difference in transfusion rates across groups. Thirty percent (14/47) of subjects who received TXA underwent an intraoperative transfusion compared with 25% (18/72) of subjects who did not receive TXA (p = 0.52).  Within those who underwent intraoperative transfusions, there were no differences detected across TXA groups with respect to packed red blood cells (p = 0.20), cell salvage RBCs (p = 0.65), or total mL RBCs per kg (p = 0.51). Thirty-two percent (15/47) of subjects who received TXA underwent a postoperative transfusion compared with 47% (34/72) of subjects who did not receive TXA, suggesting that subjects who were administered TXA had a 65% reduction in the odds of a postoperative transfusion compared with those who were not administered TXA (OR, 0.35; 95% CI, 0.13-0.93; p = 0.03). | Haematoma: Not reported  Length of stay: There was no difference detected in length of stay: TXA group median 7 days (interquartile range 5 to 12) vs non-TXA group 6 (4 to 7); p = 0.08).  Infection: Not reported  Other: Hematocrit levels were higher in the TXA group intraoperatively (mean 34.4 (SD 4.27) vs 32.1 (5.67), p = 0.047), at the end of surgery (29.6 (4.40) vs 27.4 (4.86), p = 0.04), and at the lowest levels during admission (24.2 (4.65) vs 22.4 (4.36), p = 0.03).  Complications were recorded based on the modified Clavien-Dindo system – there were no differences detected in complication rate across TXA groups (79% vs. 86%; P= 0.23). |
| Majid et al. 2015 | Gross’ formula was used to calculate total blood loss. | None for this study | Blood loss: The mean total blood loss was similar: TXA 969 mL versus no TXA 971 mL (RD: −2 mL, 95% CI: −536 mL to 531 mL, p = 0.99)  Transfusion: The proportion of children receiving blood transfusion was 42.9% in the TXA groups versus 36.1% in the non-TXA group, a statistically non-significant difference (risk difference 6.7%, 95% CI: −21.3% to 36.1%, p = 0.75) | Haematoma: Not reported  Length of stay: The duration of stay in hospital was similar between groups: TXA 8.17 days versus no TXA 7.63 (difference: 0.54 days, 95% CI: −2.37 to 3.45), p = 0.71  Infection: Two complications were recorded in the non-TXA group: one deep infection and a pressure sore.  Other: Postoperative Hemoglobin, Haematocrit, and Hb Drop. Hemoglobin (Hb) and hematocrit (Hct) were tested on postoperative day 2 unless there was a clinical need to do these earlier. Levels were similar and differences between the two groups were not statistically significant.  There were 3 complications in the tranexamic acid group (2 fragility fractures and 1 paralytic ileus). The two fractures were treated nonoperatively in a resting cast. There were no reported thromboembolic events |
| Masrouha et al. 2022 | EBL was determined by the surgeon and anesthesiologist based on fluid level in the suction  canister and number of soaked sponges | None for this study | Blood loss: There was no statistically significant difference between the TXA and non-TXA groups with regard to estimated blood loss, though there was a higher EBL in the non-TXA group without statistical significance (297 mL vs. 393 mL respectively, p = 0.254)  Transfusion: The risk for intraoperative transfusion (17% in TXA group versus 21% in non-TXA group) and postoperative transfusion (8% in TXA group versus 26% in NTXA group) showed increased occurrence in the NTXA group without statistical significance (p = 1.0 and p = 0.21 respectively). The non-TXA group also showed a tendency toward a greater number of children receiving any perioperative blood transfusion (42% vs. 21% p = 0.185). | Haematoma: Not reported  Length of stay: The TXA group had a mean length of stay of 3.8 days (range 1–12), and the non-TXA group had a mean length of stay of 4.1 days (range 2–10) (p = 0.719).  Infection: Not reported  Other: There were no reports of thromboembolic events in any of the patients who received TXA.  Postoperative haemoglobin and haematocrit were similar between the groups (p > 0.05). A significant difference was not found in the drop in haemoglobin or haematocrit from preoperative to postoperative between the two groups. The haematocrit in the TXA group dropped by 12.3%, while in the non-TXA group, it dropped by 13.3% (p = 0.627). |
| van Kouswijk et al. 2023 | Estimated blood loss will be calculated using the gravimetric method, where intraoperative blood loss is estimated by weighing surgical materials contaminated with blood and subtracting dry weights and rinse liquid. By summing the measured weight of blood and estimating the mount of mixed liquid (blood and rinse liquid) in the suction container, the blood loss is calculated with a conversion of 1g = 1mL of blood. | This project was funded by the Sophia Fund (2021WAR2248/ WO), JKF Fund (20210053), and EPOS Research Grant 2022. | Blood loss: N/A (protocol)  Transfusion: N/A (protocol) | Haematoma: N/A (protocol)  Length of stay: N/A (protocol)  Infection: N/A (protocol)  Other: N/A (protocol) |
| Zuccon 2023 | The volume of intraoperative blood loss was estimated by weighing the surgical compresses used during the procedure and subtracting the weight of dry surgical compresses. | None for this study | Blood loss: Intraoperative bleeding: TXA group = mean 194mL (SD 90) vs control group = 313 (92), p = 0.002.  Postoperative bleeding: TXA group = 96.8 (42.2) vs control group = 115 (70.3), p = 0.498.  Transfusion: 3 children in the  control group and 6 in the intervention group were transfused (p = 0.08). | Haematoma: Not reported  Length of stay: Total hospital stays for TXA group = mean 3.71 days (SD 1.35) vs control group 5.9 (SD 4.56), p = 0.049  Infection: Not reported  Other: N/A |
| OR = odds ratio; CI = confidence interval | | | | |

## Table S7: Summary of findings for studies with a primary aim related to TXA

| **Characteristic** | **Findings - spine (n = 12 studies)** | **Findings - hip (n = 10 studies)** |
| --- | --- | --- |
| *Blood loss measurement technique | *Intraoperative (9/12 = 75%):   - Anaesthetist and/or surgeon recorded a value for estimated blood loss based on factors such as surgical suction, autotransfusion system reservoirs, and weighing sponges from operative field = 5/9 (55.6%) - Measured by aspiration and by weighing the used sponges and gauze on a digital scale = 1/9 (11.1%) - Haematocrit-based formula for estimated red cell mass deficit normalised as a percentage of total blood volume and normalised blood product transfused (calculated as volume of intraoperative blood product, normalised to patient weight) = 1/9 (11.1%) - Haematocrit-based formula taking into account patient body mass and relative fluid balance = 1/9 (11.1%) - Estimated red cell mass deficit, and haemoglobin mass loss (formulae not further specified) = 1/9 (11.1%) - Determined by measuring the amount of blood collected in the suction canister and subtracting the amount of irrigation saline used, including in the sponges for packing = 1/9 (11.1%) - Estimated based on experienced surgeon and anaesthetist observation, the amount of blood in the suction canister, the weight of the lap sponges, the amount in the Cell Saver, and the first postoperative haematocrit = 1/9 (11.1%) - Blood loss into the Cell Saver system was collected in addition to weighing sponges. Calculated blood loss was then determined by subtracting the volume of heparinised saline from the total volume in the Cell Saver system, and blood-soaked sponges from averaged dry sponge weight - these two values were then summed = 1/9 (11.1%) - Calculated based on preoperative and postoperative haemoglobin, accounting for intraoperative blood products (formula not provided) = 1/9 (11.1%) - Not reported = 1/9 (11.1%)   Postoperative (3/12 = 25%):   - Measured as the volume through the drains during the first 48 postoperative hours = 1/3 (33.3%) - Estimated through monitoring of subfascial Hemovac drain outputs at the incision site = 1/3 (33.3%) - Drain debit was measured every 12 hours and recorded in the patient’s chart = 1/3 (33.3%)   Perioperative (3/12 = 25%):   - Calculated as the sum of intraoperative (measured by aspiration and by weighing the used sponges and gauze on a digital scale) and postoperative (measured as the volume through the drains during the first 48 postoperative hours) blood loss = 1/3 (33.3%) - Defined as the sum of the intraoperative bleeding (determined by measuring the amount of blood collected in the suction canister and subtracting the amount of irrigation saline used, including in the sponges for packing) and the total drain output = 1/3 (33.3%) - Not reported = 1/3 (33.3%)   Not applicable (not measured in the study) = 2/12 (16.7%) | Intraoperative (6/10 = 60%)   - EBL based on inspection of gauze and suction = 4/6 (66.7%) - Based on weight of surgical compresses, subtracting weight of dry compresses = 1/6 (16.7%) - Gravimetric method = 1/6 (16.7%)   Perioperative (5/10 = 50%)   - Haemoglobin balance method = 1/5 (20%) - Formula based on haemoglobin difference = 1/5 (20%) - Haemoglobin drop (not further specified) = 1/5 (20%) - Hematocrit-based method = 1/5 (20%) - Gross' formula [28] = 1/5 (20%) |
| Funding source | No study-specific funding = 3/12 (25%)  Not reported = 4/12 (33.3%)  Institutional/departmental = 1/12  (8.3%)  Government funding = 1/12 (8.3%)  External (non-institutional) source = 2/12 (16.7%)  Pharmaceutical company or industry = 1/12 (8.3%) | No study-specific funding = 8/10 (80%)  Not reported = 1/10 (10%)  Institutional/departmental = 0  Government funding = 0  External (non-institutional) source = 1/10 (10%)  Pharmaceutical company or industry = 0 |
| Blood loss | Intraoperative (8/12 = 75%):   - TXA use associated with reduced blood loss = 2/8 (25%) - TXA use not associated with blood loss = **6/8 (75%)   Postoperative (0)  Perioperative (2/12 = 16.7%):   - TXA use associated with reduced blood loss = 1/2 (50%) - TXA use not associated with blood loss = ***1/2 (50%)   Not applicable (3/12 = 25%):   - Not measured = 2/3 (66.7%) - RCT protocol = 1/3 (33.3%) | Intraoperative (6/10 = 60%):   - TXA use associated with reduced blood loss = 2/6 (33.3%) - TXA use not associated with blood loss = 4/6 (66.7%)   Postoperative (2/10 = 20%):   - TXA use not associated with blood loss = 2/2 (100%)   Perioperative (3/10 = 30%):   - TXA use associated with reduced blood loss = 2/3 (66.7%) - TXA use not associated with blood loss = 1/3 (33.3%)   N/A - RCT protocol (1/10 = 10%) |
| Transfusion | Intraoperative (3/12 = 25%)   - ****TXA use not associated with transfusion rate = 1/3 (33.3%) - Antifibrinolytic use not associated with transfusion rate = 1/3 (33.3%) - Unable to determine association from information provided in record (case series) = 1/3 (33.3%)   Postoperative (2/12 = 16.7%)   - TXA use not associated with transfusion rate = 1/2 (50%) - Antifibrinolytic use not associated with transfusion rate = 1/2 (50%)   Perioperative (3/12 = 25%)   - TXA use associated with reduced transfusion rate = 2/3 (66.7%) - TXA use not associated with transfusion rate = 1/3 (33.3%) | Intraoperative (4/10 = 40%)   - TXA use not associated with transfusion = 4 (100%)   Postoperative (3/10 = 30%)   - TXA use not associated with transfusion = ⅔ (66.7%)   Perioperative (7/10 = 70%)   - TXA use associated with reduction in transfusion = 2/7 (28.6%) - TXA use not associated with reduction in transfusion = 4/7 (57.2%) - TXA use associated with higher transfusion rate = 1/7 (14.3%)   Not reported = 1/10 (10%)  N/A (protocol) = 1/10 (10%) |
| *some studies used more than one technique; **one study compared TXA to aprotinin, one study compared TXA to aminocaproic acid; ****compared TXA with aprotinin; ****compared TXA with aminocaproic acid | | |

## Table S8: Findings of studies with a primary aim not related to TXA

| **Record ID** | **Blood loss measurement technique** | **Funding source (external/non-institutional)** | **Main findings** | **Secondary findings** |
| --- | --- | --- | --- | --- |
| **Spine studies (AND SPINE COMPONENT OF LU 2020)** | | | | |
| Bekmez et al. 2018 | Not reported | None for this study | Blood loss: Although intraoperative antifibrinolytics (intravenous tranexamic acid) were routinely used, both groups were associated  with substantial blood loss. Posterior column osteotomy group intraoperative blood loss = mean 1488mL (SD 304), pedicle subtraction osteotomy group = 1632  (852).  Transfusion: Units transfused PCO group = mean 3.2 (SD 0.6) | Haematoma: Not reported  Length of stay: 5.7 days (range 4-7) PCO group; 6.5 (5-12) PSO group  Infection: Not reported  Other: Not reported |
| Lu et al. 2020 | Not applicable (blood loss was not measured in this study) | None for this study | Blood loss: N/A (not measured in this study)  Transfusion: No reported association between TXA use and transfusion | Haematoma: Not reported  Length of stay: SEMLS = median 7 days (range 2-16); spinal surgery = 8 (5-128)  Infection: Not reported  Other: Percutaneous endoscopic gastrostomy feeding (odds ratio (OR) 5.0 (95% confidence interval (CI) 2.2, 11.8)), epilepsy (OR 3.4 (95% CI 1.6, 7.2)), sodium valproate usage (OR 4.3 (95% CI 1.8, 10.0)), spinal fusion versus SEMLS (OR 3.5 (95% CI 1.6, 7.7)) and spinal fusion to the pelvis (OR 3.1 (95% CI 1.4, 6.6)) were all associated with intra- or post-operative transfusion (Table 3). Successively higher GMFCS levels were also associated with increasingly higher odds of transfusion (relative odds 3.9 (95% CI 0.4, 41.0), 18.8 (95% CI 2.2, 158.5) and 33.0 (95% CI 4.2, 260.3) for levels III, IV and V relative to levels I and II combined). Compared to surgery lasting less than 2 hours, the relative odds of transfusion was 2.6 (95% CI 0.3, 22.2) for surgeries taking 2–5 h and 45.0 (95% CI 3.4, 594.1) for those lasting longer than 5 h. Referenced against one osteotomy, the relative odds of transfusion was 6.6 (95% CI 0.8, 53.2) for two osteotomies and 80.0 (95% CI 4.2, 1525.6) for three or more osteotomies. |
| McLeod et al. 2015 | Not applicable (blood loss was not measured in this study) | Supported by grants from the Pennsylvania Department of Public Health Commonwealth Universal Research Enhancement (C.U.R.E.) Program (Health Research Formula Grant), and the Kerr Family Foundation. | Blood loss: N/A (not measured in this study)  Transfusion: There was no association between red cell transfusions and the use of TXA (OR, 1.3; P=0.4) (Table 2). | Haematoma: Not reported  Length of stay: Not reported  Infection: Not reported  Other: Antifibrinolytic use increased throughout the study period (18% to 39% for NMS procedures). |
| Abbot et al. 2014 | Not reported | None for this study | Blood loss: Unable to determine effect of TXA (case series)  Transfusion: Unable to determine effect of TXA (case series) | Haematoma: Not reported  Length of stay: Not reported  Infection: Not reported  Other: N/A |
| Bird et al. 2011 | Not applicable (blood loss was not measured in this study) | Not reported | Blood loss: N/A (survey study on strategies employed to reduce perioperative blood loss)  Transfusion: N/A (survey study on strategies employed to reduce perioperative blood loss) | Haematoma: Not reported  Length of stay: Not reported  Infection: Not reported  Other: Perioperatively, 17 centers (81%) routinely used  antifibrinolytics, all of whom use tranexamic acid.  Reported dosing regimens showed an even wider variation than previously reported, with loading doses  varying between 2 and 100 mg/kg and infusions  varying from 3 to 10 mg/kg/hr |
| Chiem et al. 2017 | Not reported | None for this study | Blood loss: Unable to determine effect of TXA (case report)  Transfusion: Unable to determine effect of TXA (case report) | Haematoma: Not reported  Length of stay: Not reported  Infection: Not reported  Other: Not reported |
| Degiorgio-Miller et al. 2013 | Not reported | Not reported | Blood loss: The use of Tranexamic acid improved intraoperative blood loss in patients fused to the sacrum (no further detail provided)  Transfusion: Not reported | Haematoma: Not reported  Length of stay: Not reported  Infection: Not reported separately for patients treated with TXA  Other: N/A |
| Dhawale et al. 2012 | Blood loss was determined by the anesthesiologist, who estimated the cumulative blood loss from the sponges, the suction canister, the cell  salvage, and the surgical drapes. | Corporate and Industry funds were received to support this work. | Blood loss: There was a significant difference between the TXA group and the other groups (EACA and NAF) with respect to the EBL, EBL/level fused.  Transfusion: There was a significant difference between the TXA group and the other groups (EACA and NAF) cell salvage transfusion.  The total transfusion requirements in the TXA group were less than those in the other groups, although this was not statistically significant. | Haematoma: Not reported  Length of stay: length of stay in days (mean (SD)): antifibrinolytic group = 10 (6.5); non-antifibrinolytic group = 14 (13.9). Not reported separately for TXA group  Infection: Not reported  Other: N/A |
| Dick et al. 2019 | Not applicable (blood loss was not measured in this study) | None for this study | Blood loss: N/A (not measured in this study)  Transfusion: Authors reported that the greatest reduction in transfusion rate was seen between 2001-2003 and 2004-2006, which followed the introduction of aprotonin and cell salvage. These results suggest that aprotonin (later tranexamic acid) and cell salvage may be particularly effective in reducing transfusion rates | Haematoma: Not reported  Length of stay: Not reported  Infection: Not reported  Other: N/A |
| Dong et al. 2021 | Intraoperative blood loss included the amount of blood in suction container and surgical sponges | None for this study | Blood loss: Unable to determine effect of TXA (case series)  Transfusion: Unable to determine effect of TXA (case series) | Haematoma: Not reported  Length of stay: Not reported  Infection: Not reported  Other: Not reported |
| Dupuis et al. 2015 | Not applicable (blood loss was not measured in this study) | Support was provided solely from institutional and departmental sources. | Blood loss: N/A (not measured in this study)  Transfusion: Unable to determine effect of TXA (case series) | Haematoma: Not reported  Length of stay: Not reported  Infection: Not reported  Other: Table 1: 29 (6.0%) in non-transfused group had neuromuscular scoliosis; 13 (5.5%) in transfused group had neuromuscular scoliosis - it appears as though this difference was not 'statistically significant' |
| Duvernay et al. 2020 | Estimated red cell mass deficit, and haemoglobin mass loss (formulae not further specified) | Not reported | Blood loss: Unable to determine effect of TXA (case series)  Transfusion: Not reported | Haematoma: Not reported  Length of stay: Not reported  Infection: Not reported  Other: Despite all patients receiving a bolus of TXA with continuous low dose infusion, multiplex results indicated a progressive increase in circulating D-dimer, becoming statistically significant at postop and persisting through POD 1. Elevations in D-dimer, the byproduct of plasmin degradation of fibrin, suggest elusive fibrinolysis despite administration of TXA. |
| Eisler et al. 2020 | Not applicable (blood loss was not measured in this study) | This work was supported by an institutional training grant from the National Institutes of Health  (T32GM008464-26) (L.E.); and the National Center for Advancing Translational Sciences, National Institutes of  Health (KL2TR001874). | Blood loss: N/A (not measured in this study)  Transfusion: When the 1192 propensity score-matched pairs were compared through conditional logistic regression, use of AF was associated with a decrease in perioperative allogeneic blood transfusion (OR 0.84, 95% CI 0.68–1.05, p = 0.119), though this finding was not statistically significant. | Haematoma: Not reported  Length of stay: Propensity score-matched pairs were similar in ICU and hospital length of stay: LOS antifibrinolytic group = mean 0.89 days (SD 2.1) vs 0.92 (2.3), mean difference = 0.029, p = 0.75  Infection: Propensity score-matched pairs were similar in rate of wound infection  Other: N/A |
| Fernandez et al. 2021 | Haemoglobin and haematocrit were measured pre- and post-operatively, after reinfusion of blood recovered from cell saver. | Not reported | Blood loss: Unable to determine effect of TXA (case series)  Transfusion: Not reported | Haematoma: N/A (case series)  Length of stay: N/A (case series)  Infection: N/A (case series)  Other: N/A (case series) |
| Gurajala et al. 2013 | Blood loss was measured by weighing the sponges and blood in the suction bottles | None for this study | Blood loss: Not reported separately for TXA vs non-TXA groups  Transfusion: Not reported separately for TXA vs non-TXA groups | Haematoma: Not reported  Length of stay: Not reported separately for TXA vs non-TXA groups  Infection: Not reported  Other: N/A |
| Jurgens et al. 2017 | Not reported | Not reported | Blood loss: Unable to determine effect of TXA (case series)  Transfusion: N/A (not reported in this study) | Haematoma: N/A (case series)  Length of stay: N/A (case series)  Infection: N/A (case series)  Other: N/A (case series) |
| Koraki et al. 2020 | Not applicable (blood loss was not measured in this study) | None for this study | Blood loss: N/A (not measured in this study)  Transfusion: Protocol group received less transfused RBCs than non-protocol group (mean 0.76 (SD 0.97) units vs 1.31 (0.5), p = 0.015) | Haematoma: Not reported  Length of stay: Not reported  Infection: Not reported  Other: N/A |
| Mihas et al. 2021 | Estimated blood loss was calculated using the blood collected in the Cell Saver and weighted soaked sponges, after taking into consideration the amount of saline irrigation used. This was recorded periodically throughout the procedure.  This was then used to calculate percentage estimated blood volume loss, where estimated blood volume was calculated as 70mL/kg. | None for this study | Blood loss: Unable to determine effect of TXA (case series)  Transfusion: Unable to determine effect of TXA (case series) | Haematoma: N/A (case series)  Length of stay: N/A (case series)  Infection: N/A (case series)  Other: N/A (case series) |
| Nellis et al. 2021 | Not applicable (blood loss was not measured in this study) | Not reported | Blood loss: N/A (not measured in this study)  Transfusion: After adjusting for institution and age, the use of antifibrinolytics was not associated with a change in the incidence of transfusion (p=0.73) | Haematoma: Not reported  Length of stay: Not reported  Infection: Not reported  Other: Patients who received any blood component had longer lengths of ICU and hospital stays as compared to those not transfused, p < 0.001 for both measures) |
| O'Donoghue et al. 2020 | This was usually calculated by weighing swabs and measuring drain output. | Not reported | Blood loss: Unable to determine effect of TXA (case series)  Transfusion: Unable to determine effect of TXA (case series) | Haematoma: N/A (case series)  Length of stay: N/A (case series)  Infection: N/A (case series)  Other: N/A (case series) |
| Schur et al. 2018 | Blood loss was estimated based on experienced surgeon and anesthesiologist  observation, the amount of blood in the suction canister, the weight of the lap sponges, and the amount in the cell saver. | None for this study | Blood loss: Not reported separately for TXA vs non-TXA groups  Transfusion: Not reported separately for TXA vs non-TXA groups | Haematoma: Not reported  Length of stay: Not reported  Infection: Not reported  Other: N/A |
| Shrader et al. 2018 | Not reported | None for this study | Blood loss: Unable to determine effect of TXA (case series)  Transfusion: Unable to determine effect of TXA (case series) | Haematoma: N/A (case series)  Length of stay: N/A (case series)  Infection: N/A (case series)  Other: N/A (case series) |
| Singh et al. 2013 | Not reported | None for this study | Blood loss: Unable to determine effect of TXA (case report)  Transfusion: Not reported | Haematoma: N/A (case report)  Length of stay: N/A (case report)  Infection: N/A (case report)  Other: N/A (case report) |
| Soini et al. 2023 | Not reported | Personal research funds were received for the following authors: VS has received grants from Vappu Uuspään säätiö, Turku University research funding, and Finnish Pediatric Research  Foundation; JS from the Clinical Research Institute HUCH, and LH funding from Finnish Paediatric Research Foundation  and Finska Läkaresällskapet. IH has received scientific funding from Industry to Institutions from Medtronic, Stryker, Nuvasive, and Cerapedics. IH has been working as  a consultant for Medtronic. Completed disclosure forms for  this article following the ICMJE template are available on the  article page, doi: 10.2340/17453674.2023.11962 | Blood loss: Unable to determine effect of TXA (case series)  Transfusion: Not reported | Haematoma: N/A (case series)  Length of stay: N/A (case series)  Infection: N/A (case series)  Other: N/A (case series) |
| Spiessberger et al. 2023 | Not reported | This study was supported in part by a grant from Depuy Synthes Spine to the Setting Scoliosis Straight Foundation in support of  Harms Study Group research | Blood loss: Not reported separately for TXA vs non-TXA groups  Transfusion: N/A (not mentioned in this study) | Haematoma: Not reported  Length of stay: Not reported for TXA vs non-TXA groups  Infection: Not reported  Other: N/A |
| Tan et al. 2018 | Not reported | Not reported | Blood loss: Not reported separately for TXA vs non-TXA groups  Transfusion: Tranexamic acid use (p = 0.036) was associated with a decrease in PRBC volume).  After accounting for patient risk and surgeon, antifibrinolytics (OR 0.44, p = 0.002) was associated with decreased PRBC transfusion. | Haematoma: Not reported  Length of stay: Not reported  Infection: Not reported  Other: N/A |
| Vasan et al. 2021 | Intraoperative blood loss = "suction apparatus, gauze/pad weight"; postoperative blood loss = drain output | Ganga Orthopedic Research and Education Fund (GOREF) | Blood loss: Unable to determine effect of TXA (case series)  Transfusion: Unable to determine effect of TXA (case series) | Haematoma: N/A (case series)  Length of stay: N/A (case series)  Infection: N/A (case series)  Other: N/A (case series) |
| Vrbica et al. 2023 | Not reported | The project was supported by Specific University Research provided  by MSMT (MUNI/A/1336/2022) and by the Ministry of Health, Czech Republic—  conceptual development of research organisation (FNBr, 65269705). The project  was supported by the national budget through MEYS, RI CZECRIN (LM2018128) | Blood loss: N/A (protocol)  Transfusion: N/A (protocol) | Haematoma: N/A (protocol)  Length of stay: N/A (protocol)  Infection: N/A (protocol)  Other: N/A (protocol) |
| Fernandes 2020 | Authors specified that estimated blood loss, blood loss per blood volume (weight multiplied by 0.70), and blood loss per instrumented levels were considered as relevant outcomes. No further details were provided. | Not reported | Blood loss: Blood losses per blood volume pre multimodal program = mean 21.4% (SD 13.3) vs post multimodal program = 16.6 (14.4), p = 0.026  Transfusion: Transfusion rate pre multimodal program = 98.7% (52) vs post multimodal program = 66%(47), p < 0.001 | Haematoma: Not reported  Length of stay: Not reported  Infection: Not reported  Other: N/A |
| **Hip studies (AND HIP COMPONENT OF LU 2020)** | | | | |
| Adler et al. 2022 | Not applicable (blood loss was not measured in this study) | None for this study | Blood loss: N/A (not measured in this study)  Transfusion: We observed reductions in transfusions among those receiving antifibrinolytics in both treatment groups. These unadjusted reductions in transfusions among those receiving antifibrinolytics were not statistically significant in the Neuromuscular/Syndromic patients (p = 0.15) | Haematoma: Not reported  Length of stay: Non-transfusion group = mean 4.1 days (SD 3.2) vs transfusion = 4.0 (3.8), p = 0.92  Infection: Not reported  Other: There were no adverse events from tranexamic acid  identified.  There was no difference in antifibrinolytic use between DD and NM patients. Adjusted factors associated with increased odds of intraoperative transfusion were NM status (vs DD status) (Odds Ratio (OR) =2.96, 95% CI (1.76, 5.00) and the number of osteotomies performed (OR = 1.82 per osteotomy, 95% CI (1.40, 2.35). Adjusted factors that reduced the odds of  transfusion were the use of antifibrinolytics (OR = 0.35, 95% CI (0.17, 0.71) and regional  anesthesia (OR = 0.52, 95%CI (0.29, 0.94). The model had both good discrimination (area  under ROC curve =0.75) and calibration (Hosmer-Lemeshow statistic = 8.35, p = 0.30). |
| Lu et al. 2020 | Not applicable (blood loss was not measured in this study) | None for this study | Blood loss: N/A (not measured in this study)  Transfusion: No reported association between TXA use and transfusion | Haematoma: Not reported  Length of stay: SEMLS = median 7 days (range 2-16); spinal surgery = 8 (5-128)  Infection: Not reported  Other: Percutaneous endoscopic gastrostomy feeding (odds ratio (OR) 5.0 (95% confidence interval (CI) 2.2, 11.8)), epilepsy (OR 3.4 (95% CI 1.6, 7.2)), sodium valproate usage (OR 4.3 (95% CI 1.8, 10.0)), spinal fusion versus SEMLS (OR 3.5 (95% CI 1.6, 7.7)) and spinal fusion to the pelvis (OR 3.1 (95% CI 1.4, 6.6)) were all associated with intra- or post-operative transfusion (Table 3). Successively higher GMFCS levels were also associated with increasingly higher odds of transfusion (relative odds 3.9 (95% CI 0.4, 41.0), 18.8 (95% CI 2.2, 158.5) and 33.0 (95% CI 4.2, 260.3) for levels III, IV and V relative to levels I and II combined). Compared to surgery lasting less than 2 hours, the relative odds of transfusion was 2.6 (95% CI 0.3, 22.2) for surgeries taking 2–5 h and 45.0 (95% CI 3.4, 594.1) for those lasting longer than 5 h. Referenced against one osteotomy, the relative odds of transfusion was 6.6 (95% CI 0.8, 53.2) for two osteotomies and 80.0 (95% CI 4.2, 1525.6) for three or more osteotomies." |

## Table S9: Summary of findings for studies with a primary aim not related to TXA

| **Characteristic** | **Findings - spine (n = 29)** | **Findings - hip (n = 2)** |
| --- | --- | --- |
| *Blood loss measurement technique | Intraoperative (11/29 = 37.9%):   - Blood loss was determined by the anesthesiologist, who estimated the cumulative blood loss from the sponges, the suction canister, the cell salvage, and the surgical drapes = 1/11 (9.1%) - Blood loss was estimated based on experienced surgeon and anesthesiologist observation, the amount of blood in the suction canister, the weight of the lap sponges, and the amount in the cell saver = 1/11 (9.1%) - Intraoperative blood loss included the amount of blood in suction container and surgical sponges = 4/11 (36.4%) - Estimated red cell mass deficit, and haemoglobin mass loss (formulae not further specified) = 1/11 (9.1%) - Haemoglobin and haematocrit were measured pre- and post-operatively, after reinfusion of blood recovered from cell saver = 1/11 (9.1%) - Estimated blood loss was calculated using the blood collected in the Cell Saver and weighted soaked sponges, after taking into consideration the amount of saline irrigation used. This was recorded periodically throughout the procedure = 1/11 (9.1%) - Estimated blood loss was calculated using the blood collected in the Cell Saver and weighted soaked sponges, after taking into consideration the amount of saline irrigation used. This was recorded periodically throughout the procedure. This was then used to calculate percentage estimated blood volume loss, where estimated blood volume was calculated as 70mL/kg = 1/11 (9.1%) - Authors specified that estimated blood loss, blood loss per blood volume (weight multiplied by 0.70), and blood loss per instrumented levels were considered as relevant outcomes. No further details were provided = 1/11 (9.1%)   Postoperative (1/29 = 3.4%):   - Measuring drain output = 1/1 (100%)   Perioperative (0)  Not applicable (not measured in the study) = 8/29 (27.6%)  Not reported = 11/29 (37.9%) | Not applicable (not measured in the study) = 2/2 (100%) |
| **Funding source | No study-specific funding = 12/29 (41.4%)  Not reported = 9/29 (31.0%)  Institutional/departmental = 3/29 (10.3%)  Government funding = 3/29 (10.3%)  External (non-institutional) source = 1/29 (3.4%)  Pharmaceutical company or industry = 3/29 (10.3%) | No study-specific funding = 2/2 (100%) |
| Blood loss | Intraoperative (17/29 = 58.6%):   - TXA use associated with reduced blood loss = ****3/17 (17.6%) - Unable to determine association from information provided in record (case series) = 14/17 (83.4%)   Postoperative (0)  Perioperative (3/29 = 10.3%):   - Unable to determine association from information provided in record (case series) = 3/3 (100%)   Not applicable (9/29 = 31.0%)   - Not measured = 8/9 (88.9%) - RCT protocol = 1/9 (11.1%) | Not applicable (not measured in the study) = 2/2 (100%) |
| ***Transfusion | Intraoperative (8/29 = 27.6%)   - ****TXA use associated with reduced transfusion rate = 2/8 (25%) - Unable to determine association based on information provided in record (case series) = 5/8 (62.5%) - Unable to determine association based on information provided in record (insufficient information) = 1/8 (12.5%)   Postoperative (1/29 = 3.4%)   - TXA use not associated with transfusion rate = 1/1 (100%)   Perioperative (13/29 = 44.8%)   - ****TXA use associated with reduced transfusion rate = 3/13 (23.1%) - TXA use not associated with transfusion = 1/13 (7.7%) - Antifibrinolytic not associated with transfusion = 2/13 (15.4%) - Unable to determine association based on information provided in recorded (case series) = 5/13 (38.5%) - Unable to determine association based on information provided in record (insufficient information) = 2 (15.4%)   Not applicable (3/29 = 10.3%)   - RCT protocol = 1/3 (33.3%) - Not measured in the record = 2/3 (66.7%)   Not reported (5/29 = 17.2%) | Intraoperative (1/2 = 50%)   - Antifibrinolytic not associated with transfusion = 1/1 (100%)   Postoperative (1/2 = 50%)   - TXA use not associated with transfusion rate = 1/1 (100%) |
| Full detail of findings, in as much detail as could be extracted from included studies, is available in the Supplementary File; *some studies used more than one technique; **some studies had more than one source of funding; ***one study reported intraoperative and perioperative transfusion separately; ****one study included TXA as part of a blood loss reduction protocol, and the protocol was analysed for its impact on transfusion | | |

# Table S10: Findings of grey literature search

| **Source** | **Date accessed** | **Number of records retrieved and screened** | **Relevant records included** |
| --- | --- | --- | --- |
| WHO International Clinical Trials Registry Platform (ICTRP) | 16/05/2024 | 77 | 1 -> 0 |
| clinaltrials.gov | 16/05/2024 | 102 | 4 -> 0 |
| New York Academy of Medicine Grey Literature Report | 16/05/2024 | 8 | 0 |
| Open Science Framework | 16/05/2024 | 358 | 0 |
| ProQuest | 17/5/2024 | 109 | 9 -> 2 |
| Dimensions | 18/5/2024 | Datasets: 173  Grants: 186  Policy:  372 | Datasets: 0 Grants: 0  Policy: 0 |
| Trove | 17/5/2024 | Research & Reports: 203  Book & Libraries: 275 | Research & Reports: 0  Book & Libraries: 3 -> 0 |
| WHO IRIS | 18/05/2024 | 128 | 0 |
| General Google Search | 18/05/2024 | ~ 70 result for each term | 0 |

# Figure S1: Publications on TXA in children with cerebral palsy undergoing hip or spine surgery


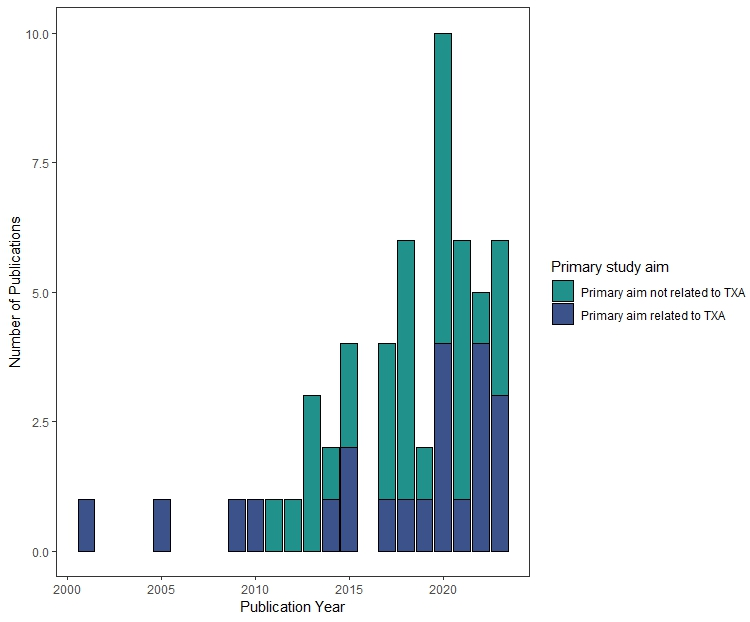

Supplement: Supplementary file 1 — Supplementary Material 1. Search strategies. MEDLINE (Ovid). EMBASE (Ovid). Web of Science Core Collection. Google Scholar (advanced search). Table S1: Grey literature. Table S2: Individual study characteristics. Table S3: Study characteristics – summarised. Table S4: Patient and intervention characteristics – details of individual studies. Table S5: Patient and intervention characteristics – summarised. Table S6: Findings of studies with a primary aim related to TXA. Table S7: Summary of findings for studies with a primary aim related to TXA. Table S8: Findings of studies with a primary aim not related to TXA. Table S9: Summary of findings for studies with a primary aim not related to TXA. Table S10: Findings of grey literature search. Figure S1: Publications on TXA in children with cerebral palsy undergoing hip or spine surgery. [file 13643_2024_2734_MOESM1_ESM.docx]
